# Disabling COMPASS Promoter Licensing Shifts HDAC‐Inhibited Gastric Cancer Toward Sustained Transcriptional Suppression During Recovery

**DOI:** 10.1002/advs.78255

**Published:** 2026-10-15

**Authors:** Jintian Song, Hui Yu, Jianping Jiang, Zhihui Jiang, Zhongshi Hong, Xu Zhang, Luchuan Chen, Shenghong Wei, Yi Wang

**Affiliations:** ^1^ Department of Gastrointestinal Oncology Clinical Oncology School of Fujian Medical University Fujian Cancer Hospital Fujian Branch of Fudan University Shanghai Cancer Center Fuzhou Fujian People's Republic of China; ^2^ Department of Pharmacy Clinical Oncology School of Fujian Medical University Fujian Cancer Hospital Fujian Branch of Fudan University Shanghai Cancer Center Fuzhou Fujian People's Republic of China; ^3^ Department of Gastric Surgery Clinical Oncology School of Fujian Medical University Fujian Cancer Hospital Fujian Branch of Fudan University Shanghai Cancer Center Fuzhou Fujian People's Republic of China; ^4^ Department of General Surgery The Second Affiliated Hospital of Fujian Medical University Quanzhou Fujian People's Republic of China; ^5^ University of Alabama at Birmingham Birmingham Alabama USA

**Keywords:** apoptosis, biology, cancer cell, cancer research, cancer, chromatin remodeling, compass, epigenetics, histone, in vivo, programmed cell death

## Abstract

Histone deacetylase inhibitors (HDACi) broadly increase chromatin acetylation but have limited and variable activity in solid tumors. To define the epigenetic buffering axis that sustains promoter output during HDAC inhibition in gastric cancer. We integrated pharmacologic profiling across a molecularly diverse gastric cancer panel with an epigenetic‐focused pooled CRISPR‐Cas9 screening library, domain‐resolved genetic perturbation, structure‐guided WIN‐pocket targeting, CCK8 time‐course proliferation assays, and pulse‐washout clonogenic recovery assays. Chromatin and transcriptional consequences were interrogated by assessing COMPASS integrity and chromatin association, promoter‐proximal H3K4me3 dynamics, data‐derived ATAC‐seq accessibility, and transcriptional output. WDR5 abundance and baseline H3K4me3 levels were associated with sensitivity to combined HDAC and WDR5 WIN‐pocket inhibition. Multiple orthogonal approaches identified the WDR5 WIN pocket as a key interface supporting chromatin‐associated COMPASS and promoter‐proximal H3K4me3 under the tested conditions. WIN‐pocket disruption reduced chromatin recovery of COMPASS‐associated components and attenuated H3K4me3 at selected growth‐linked promoters. This produced a state with elevated H3K27 acetylation, attenuated H3K4me3, and reduced transcriptional output; data‐derived ATAC‐enriched regions retained a centered accessibility signal, although the mean peak amplitude was lowest in the combination. HDAC inhibitor response is conditioned by promoter licensing via the WDR5‐COMPASS H3K4 methylation axis.

## Graphical abstract

Combined inhibition of the WDR5 WIN pocket and HDAC activity converts SAHA‐induced hyperacetylation into a promoter state with reduced WDR5–COMPASS association and H3K4me3. MYC/E2F‐linked nascent transcription and biosynthetic recovery are suppressed, while apoptosis and senescence‐like features increase, resulting in sustained growth inhibition in gastric cancer cells and xenografts within the tested recovery window.

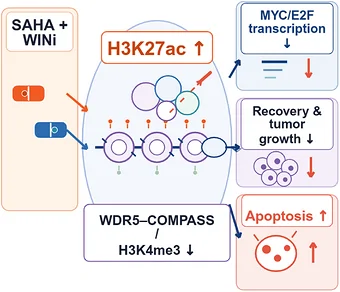

## 1 Introduction

Gastric cancer remains a major global health burden, with ∼1.1 million new cases and ∼0.8 million deaths worldwide each year, ranking among the top causes of cancer mortality despite incremental advances in systemic therapy [1, 2]. Given the pervasive epigenetic dysregulation in gastric tumors, histone deacetylase (HDAC) inhibitors have been explored as therapeutic agents. Vorinostat (SAHA), the first HDAC inhibitor approved by the U.S. FDA, established clinical proof‐of‐concept for the class, but activity in solid tumors has generally been modest, motivating rational combinations informed by mechanism [3, 4, 5].

Promoter‐proximal H3K4 methylation deposited by SET1/MLL (KMT2) COMPASS complexes is a central determinant of transcriptional competence. WDR5 is a WD40 scaffold that binds conserved WIN motifs on MLL/SET1 subunits and nucleates the RBBP5–ASH2L–DPY30 catalytic ensemble; structural work shows a high‐affinity arginine‐binding WIN pocket on WDR5 that is essential for complex assembly [6, 7]. Small‐molecule WIN‐site antagonists disrupt WDR5–MLL binding and blunt H3K4 methylation outputs in cells, identifying a tractable protein–protein interaction surface for chemical interrogation [8, 9].

Beyond its structural role in COMPASS, WDR5 cooperates with MYC/N‐MYC at active chromatin to specify and amplify growth‐linked transcriptional programs. Genetic and genomic studies show that WDR5 is required for efficient MYC recruitment and expression of MYC target genes, linking a COMPASS scaffold to oncogenic transcription and cell‐cycle progression [10, 11]. These observations suggest that constraining WDR5—particularly via the WIN pocket—could impair E2F/MYC‐driven transcription and expose vulnerabilities to HDAC blockade.

Here, we combined epigenetic‐focused pooled CRISPR knockout screens under ±SAHA with domain‐tiling CRISPR across WDR5, engineered precise domain deletions (WIN_del/WBM_del), and quantitative functional readouts, including CCK8 time‐course proliferation assays, dose‐response with growth‐rate [GR] metrics [12, 13, 14], competitive fitness and pharmacologic synergy matrices [15, 16], to identify epigenetic regulators that condition HDAC‐inhibitor response in gastric cancer. To anchor the mechanism, we integrated steady‐state RNA‐seq, 5‐EU nascent RNA capture, EU/OPP biosynthesis profiling, ChIP‐qPCR, CUT&Tag promoter chromatin profiling, ATAC‐seq, and xenograft pharmacodynamic assays. The central model tested is that HDAC inhibition increases acetylation while transcriptional output remains buffered by WDR5‐COMPASS‐dependent H3K4me3; disabling this licensing axis reduces H3K4me3 and transcriptional output without requiring complete loss of accessible chromatin.

## 2 Results

### 2.1 Baseline WDR5 Abundance is Associated with Context‐Dependent Sensitivity to WINi Plus SAHA in Gastric Cancer

To characterize heterogeneity in the response of gastric cancer cells to HDAC and WDR5 WIN‐site inhibition, we profiled a panel of gastric cancer cell lines representing intestinal, diffuse/signet‐ring cell carcinoma (SRCC), microsatellite instability (MSI), and EBV‐positive subtypes, together with GES‐1 cells as a non‐tumor gastric epithelial reference. All cell lines included in the panel were annotated for short tandem repeat authentication and mycoplasma testing (Figure 1a). Growth rate‐corrected single‐agent profiling revealed substantial variation in both GR50 and GR_AOC values for vorinostat (SAHA) and the WDR5 WIN‐site inhibitor (WINi), indicating that neither agent produced a uniform response across the panel (Figure 1b). A comparable range of single‐agent responses was observed in the biomarker‐profiled subset of cell lines (Figure 1h).

**FIGURE 1:**
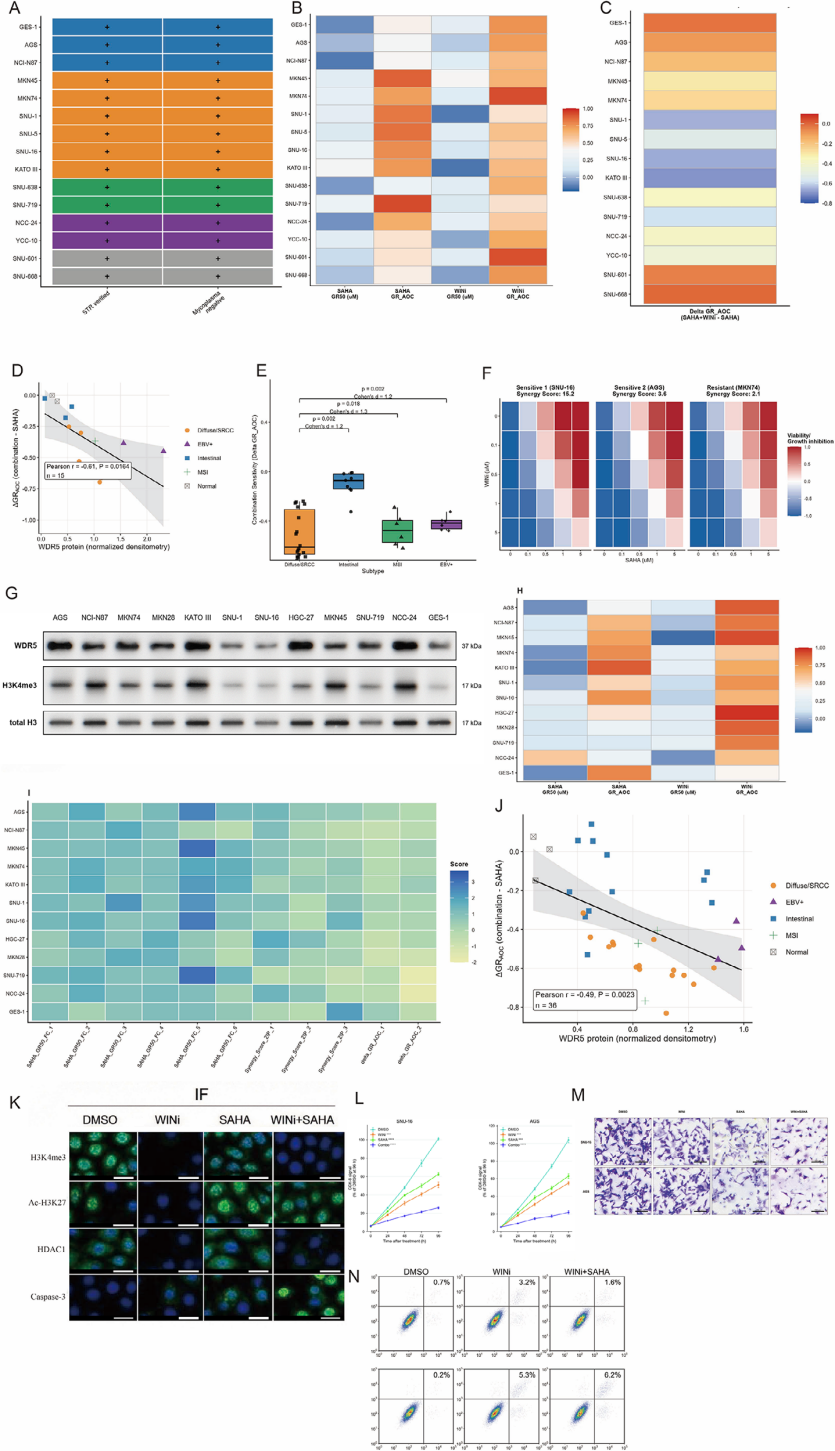
Baseline WDR5 abundance is associated with context‐dependent sensitivity to WINi plus SAHA in gastric cancer. (a) Gastric cancer cell‐line panel annotated by molecular subtype and quality‐control status, including short tandem repeat verification and mycoplasma testing. GES‐1 was included as a non‐tumor gastric epithelial reference. (b) Heatmap of normalized single‐agent SAHA and WINi responses across the full cell‐line panel, summarized using GR50 and GR_AOC metrics. (c) Change in GR_AOC following addition of WINi to SAHA, calculated as ΔGR_AOC = GR_AOC(SAHA + WINi) – GR_AOC(SAHA). (d) Correlation between baseline WDR5 protein abundance and ΔGR_AOC across cell‐line means. Pearson r = −0.61, P = 0.0164, *n* = 15. (e) Distribution of ΔGR_AOC values across diffuse/SRCC, intestinal, MSI, and EBV‐positive gastric cancer subtypes. (f) Representative SAHA‐WINi checkerboard response matrices for SNU‐16, AGS, and MKN74 cells. The reported interaction scores were 15.2, 3.6, and 2.1, respectively. (g) Immunoblot analysis of basal WDR5 and H3K4me3 abundance across the indicated cell lines. WDR5 is labeled at 37 kDa, and H3K4me3 and total histone H3 are labeled at 17 kDa. (h) Heatmap of normalized SAHA and WINi GR50 and GR_AOC values in the biomarker‐profiled cell‐line subset. (i) Heatmap summarizing replicate‐level pharmacologic response features across the biomarker‐profiled subset, including SAHA GR50 fold‐change values, ZIP synergy scores, and ΔGR_AOC measurements. (j) Replicate‐level correlation analysis between baseline WDR5 protein abundance and ΔGR_AOC. Pearson r = −0.49, P = 0.0023, *n* = 36. (k) Representative immunofluorescence images of H3K4me3, H3K27ac (labeled Ac‐H3K27 in the panel), HDAC1, and caspase‐3 after treatment with DMSO, WINi, SAHA, or WINi plus SAHA. Marker signal is shown with DAPI counterstain. (l) CCK8 time‐course proliferation curves in AGS and SNU‐16 cells treated with DMSO, WINi, SAHA, or WINi plus SAHA. Relative CCK8 signal is plotted at 0, 24, 48, 72, and 96 h and normalized to the corresponding 96‐h control signal. (m) Representative transwell migration images for SNU‐16 and AGS cells under the indicated treatment conditions. Scale bars, 100 µm. (n) Representative Annexin V/PI flow‐cytometry plots after DMSO, WINi, or WINi plus SAHA treatment. Percentages denote the upper‐right Annexin V‐positive/PI‐positive population in each plot. Statistical significance is indicated as follows: ^*^ *p* < 0.05, ^**^ *p* < 0.01, ^***^ *p* < 0.001, and ^****^ *p* < 0.0001.

We next assessed whether sublethal WINi exposure altered the response to SAHA. The resulting change in GR_AOC, calculated as the response to SAHA plus WINi relative to SAHA alone, varied markedly among cell lines (Figure 1c). Several models displayed a pronounced negative ΔGR_AOC, consistent with increased sensitivity under combined treatment, whereas other models showed little change or a positive shift. Thus, the effect of WIN‐site inhibition on SAHA response was context‐dependent rather than universal. Representative checkerboard matrices further illustrated this heterogeneity: SNU‐16 cells showed a comparatively strong interaction score of 15.2, whereas AGS and MKN74 cells showed substantially lower scores of 3.6 and 2.1, respectively (Figure 1f). The complementary heatmap of replicate‐level pharmacologic response features, including SAHA GR50 fold‐change values, ZIP synergy scores, and ΔGR_AOC measurements, similarly supported a continuous spectrum of responses across the profiled models rather than a binary sensitive–resistant classification (Figure 1i).

To identify features associated with this differential response, we examined basal WDR5 and H3K4me3 abundance in a subset of gastric cancer models. Immunoblotting showed considerable variation in WDR5 protein and global H3K4me3 levels across the cell‐line panel, with total histone H3 serving as the loading control for H3K4me3 measurements (Figure 1g). Baseline WDR5 abundance was inversely correlated with ΔGR_AOC in the cell‐line mean analysis (Figure 1d), indicating that models with higher WDR5 expression tended to exhibit a greater negative shift in the combination‐response metric. A replicate‐level analysis reproduced this association, although with a more moderate effect size (Figure 1j). Subtype stratification showed that diffuse/SRCC models generally exhibited lower ΔGR_AOC values than intestinal models, although responses overlapped with those of MSI and EBV‐positive models (Figure 1e). These data therefore nominate basal WDR5 abundance as a candidate response‐associated marker, while indicating that molecular subtype alone does not fully determine combination sensitivity.

We then examined treatment‐associated cellular phenotypes in selected models. Representative immunofluorescence images showed treatment‐associated changes in nuclear H3K4me3, H3K27ac, HDAC1, and caspase‐3 staining under DMSO, WINi, SAHA, and WINi plus SAHA treatment (Figure 1k). Because these measurements were obtained by immunofluorescence, they reflect changes in global nuclear signal and do not establish promoter‐specific redistribution. CCK8 time‐course assays in SNU‐16 and AGS cells showed progressive expansion of the DMSO control signal over 0–96 h, partial suppression by WINi or SAHA alone, and the lowest relative CCK8 signal in the WINi plus SAHA group (Figure 1l). A similar treatment ranking was observed in transwell migration assays in SNU‐16 and AGS cells, in which combination‐treated cultures showed the lowest density of migrated cells in the representative fields (Figure 1m).

Representative Annexin V/PI plots showed modest, cell‐line‐dependent changes in the upper‐right Annexin V‐positive/PI‐positive fraction (Figure 1n).

**Epigenetic‐focused pooled CRISPR screening reveals shared and context‐specific modifiers of SAHA response, while WIN‐site inhibition reduces WDR5‐COMPASS chromatin association and cellular fitness**.

To compare epigenetic regulators that modify HDAC inhibitor response in gastric cancer models with different baseline drug sensitivities, we first characterized the response of AGS and MKN45 cells to vorinostat (SAHA). MKN45 cells displayed a higher reported SAHA IC50 than AGS cells under the assay conditions, indicating lower baseline sensitivity in the MKN45 model (Figure 2a,b). We subsequently performed parallel epigenetic‐focused pooled CRISPR‐Cas9 loss‐of‐function screens in the two cell lines. Following sgRNA‐library transduction, cells were maintained for 14 days under DMSO or SAHA selection, and treatment‐associated changes in sgRNA abundance were determined by sequencing (Figure 2c).

**FIGURE 2:**
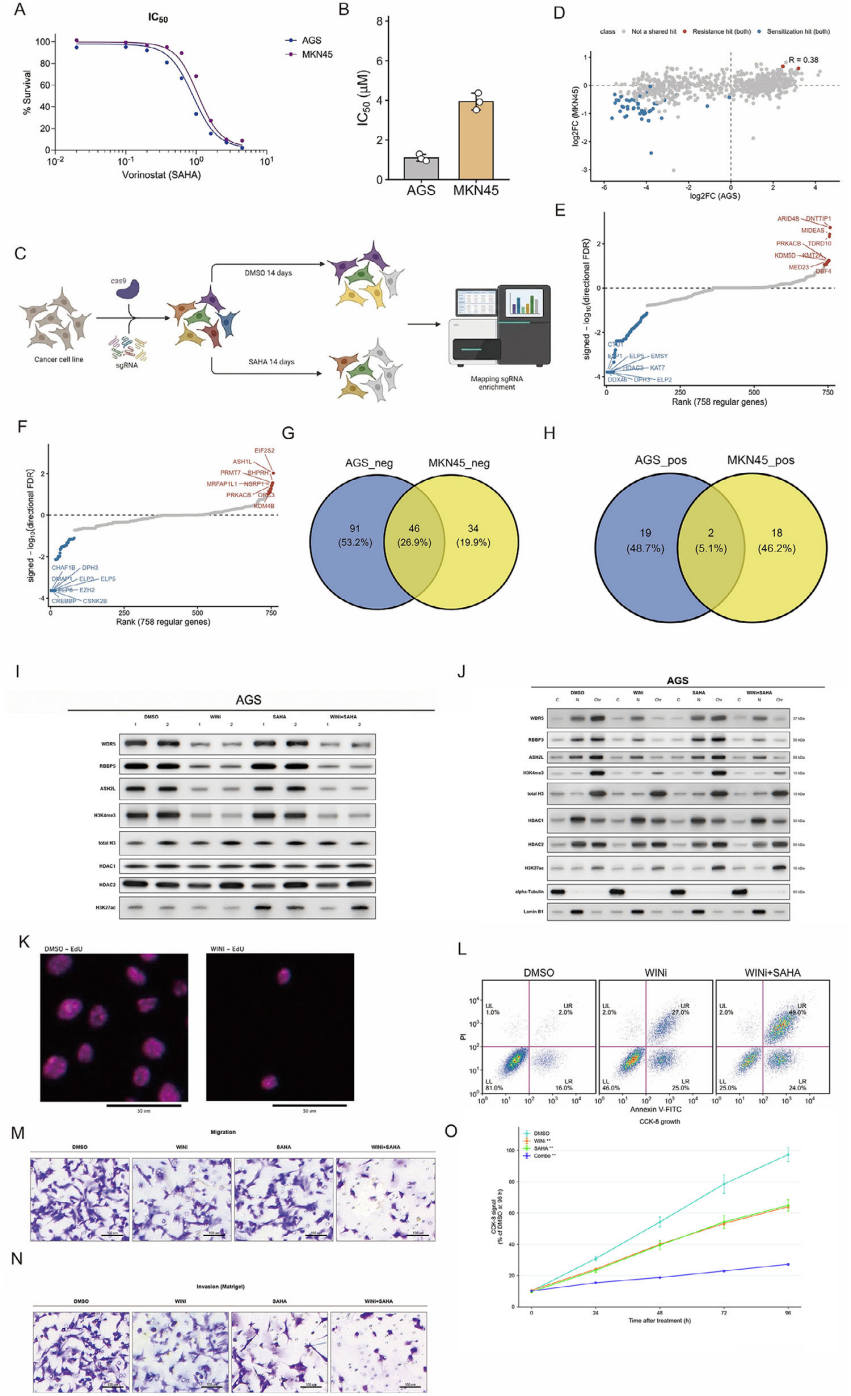
Epigenetic‐focused pooled CRISPR screening reveals shared and context‐specific modifiers of SAHA response, while WIN‐site inhibition reduces WDR5‐COMPASS chromatin association and cellular fitness. (a) SAHA dose–response curves in AGS and MKN45 gastric cancer cells. Cell survival is plotted against SAHA concentration. (b) Summary of the reported SAHA IC50 values in AGS and MKN45 cells. Individual measurements are overlaid on the summary bars; bars show mean ± s.e.m. for the plotted replicate measurements. (c) Schematic of the parallel epigenetic‐focused pooled CRISPR‐Cas9 loss‐of‐function screening workflow. Cas9‐expressing AGS and MKN45 cells were transduced with an epigenetic‐focused sgRNA library containing 24 615 sgRNAs per comparison, including 758 regular target genes represented by 30 sgRNAs per gene and safe‐targeting control guides (AAVS1 = 1671; CCR5 = 78; ROSA26 = 126). Cells were divided into DMSO and SAHA treatment arms, maintained under selection for 14 days, and analyzed by sequencing of sgRNA abundance. (d) Cross‐cell‐line comparison of gene‐level SAHA‐vs.‐DMSO log2 fold changes in AGS and MKN45 screens. Gray points indicate genes that were not classified as shared hits; blue points indicate candidate sensitization hits identified in both models; red points indicate candidate resistance hits identified in both models. The reported correlation coefficient was R = 0.38. (e, f) Ranked signed‐significance plots for the AGS and MKN45 screens, respectively. Negative scores indicate candidate sensitization genes depleted under SAHA selection, whereas positive scores indicate candidate resistance genes enriched under SAHA selection. Selected high‐ranking candidates are labeled. (g) Venn diagram showing overlap of negative, sensitization‐associated candidate genes between the AGS and MKN45 screens. Forty‐six candidates were shared between the two models. (h) Venn diagram showing overlap of positive, resistance‐associated candidate genes between the AGS and MKN45 screens. Two candidates were shared between the two models. (i) Immunoblot analysis of WDR5, RBBP5, ASH2L, H3K4me3, HDAC1, HDAC2, and H3K27ac in AGS cells treated with DMSO, WINi, SAHA, or WINi plus SAHA. Total histone H3 is shown as the reference for histone‐mark abundance. The blots show reduced WDR5–COMPASS and H3K4me3 signals in WINi‐containing conditions and increased H3K27ac in SAHA‐containing conditions. (j) Subcellular fractionation of AGS cells after treatment with DMSO, WINi, SAHA, or WINi plus SAHA. C, cytosolic fraction; N, soluble nuclear fraction; Chr, chromatin‐bound fraction. α‐Tubulin, Lamin B1, and histone H3 were used as markers of the cytosolic, soluble nuclear, and chromatin fractions, respectively. The distributions of WDR5, RBBP5, ASH2L, H3K4me3, HDAC1, HDAC2, and H3K27ac were examined across the three biochemical compartments. (k) Representative EdU incorporation images comparing DMSO‐ and WINi‐treated cells. EdU‐positive nuclei are shown in magenta; DAPI‐stained nuclei are shown in blue. Scale bars, 50 µm. (l) Representative Annexin V–FITC/PI flow‐cytometry profiles after DMSO, WINi, or WINi plus SAHA treatment. Percentages indicate the proportion of cells in each quadrant. Annexin V‐positive cells are represented by the lower‐right and upper‐right quadrants. (m) Representative transwell migration images after treatment with DMSO, WINi, SAHA, or WINi plus SAHA. Scale bars, 100 µm. (n) Representative Matrigel invasion images after treatment with DMSO, WINi, SAHA, or WINi plus SAHA. Scale bars, 100 µm. (o) CCK8 time‐course proliferation curve after treatment with DMSO, WINi, SAHA, or WINi plus SAHA. Relative CCK8 signal is plotted at 0, 24, 48, 72, and 96 h and normalized to the corresponding 96‐h control signal. Statistical significance is indicated as follows: ^*^ *p* < 0.05, ^**^ *p* < 0.01, ^***^ *p* < 0.001, and ^****^ *p* < 0.0001.

The epigenetic‐focused library contained 24 615 sgRNAs in each AGS or MKN45 comparison. The library targeted 758 epigenetic/chromatin‐regulatory genes with 30 sgRNAs per regular target gene, together with safe‐targeting control guides. Comparison of gene‐level SAHA‐vs.‐DMSO log2 fold changes revealed moderate concordance between the AGS and MKN45 screens (R = 0.38; Figure 2d), indicating that the epigenetic determinants of SAHA response comprised both shared and cell‐line‐specific components. Ranked signed‐significance plots identified candidate sensitization genes that were depleted under SAHA selection and candidate resistance genes that were enriched under SAHA selection in each model (Figure 2e,f). The overlap was greater among negative, sensitization‐associated candidates: 46 negative candidates were shared between AGS and MKN45, whereas only two positive, resistance‐associated candidates were common to both screens (Figure 2g,h). Several labeled candidates were associated with chromatin or transcriptional regulation, providing a rationale for targeted pharmacologic examination of WDR5‐COMPASS function.

We next examined the biochemical effects of pharmacologic WIN‐site inhibition in AGS cells. Immunoblotting showed that WINi‐containing conditions were associated with lower WDR5, RBBP5, ASH2L, and H3K4me3 signals than DMSO or SAHA alone (Figure 2i). In contrast, SAHA‐containing conditions produced a marked increase in H3K27 acetylation, whereas total histone H3 was broadly maintained. HDAC1 and HDAC2 did not exhibit a uniform treatment‐dependent loss. The combination condition therefore displayed concurrent reduction of H3K4me3 and elevation of H3K27ac at the bulk‐cell level.

Subcellular fractionation was validated by enrichment of α‐tubulin, Lamin B1 and histone H3 in the cytosolic, soluble nuclear and chromatin fractions, respectively (Figure 2j). Under DMSO or SAHA treatment, WDR5, RBBP5, and ASH2L were readily recovered from the chromatin fraction. WINi‐containing conditions showed lower chromatin‐fraction recovery of these proteins, with detectable signal retained in the soluble nuclear fraction. Chromatin‐associated H3K4me3 was similarly reduced under WINi‐containing conditions, whereas chromatin‐associated H3K27ac was increased in SAHA‐containing conditions. HDAC1 and HDAC2 remained predominantly nuclear and chromatin‐associated and did not show a consistent treatment‐dependent depletion.

At the cellular level, EdU imaging comparing DMSO and WINi alone showed fewer EdU‐positive nuclei after WIN‐site inhibition, consistent with reduced DNA synthesis or S‐phase entry (Figure 2k). Because SAHA and the combination were not included in this experiment, the EdU panel does not demonstrate an additional effect of SAHA. Representative Annexin V/PI profiles showed an increased Annexin V‐positive fraction after WINi treatment and a further increase in the WINi plus SAHA condition relative to DMSO (Figure 2l).

In CCK8 time‐course assays, WINi and SAHA each slowed proliferative expansion, and the combination condition produced the lowest relative CCK8 signal over the 0–96 h measurement window (Figure 2o). Representative transwell migration and Matrigel invasion fields similarly showed fewer recovered cells after either monotherapy and the lowest cell density after combined treatment (Figure 2m,n).

### 2.2 An Intact WDR5 WIN Pocket Supports H3K4me3 Maintenance and Proliferative Fitness and Shapes the Chromatin Response to Combined WIN and HDAC Inhibition

To define the structural requirement for WDR5‐dependent chromatin regulation, we depleted WDR5 using two independent sgRNAs and performed rescue experiments with sgRNA‐resistant WDR5‐WT or a WIN‐pocket‐defective WDR5 mutant. Both sgRNAs substantially reduced WDR5 protein abundance and were accompanied by a marked reduction in global H3K4me3, whereas total histone H3 and β‐actin remained broadly stable (Figure 3a). Re‐expression of WDR5‐WT restored H3K4me3 toward the level observed in non‐targeting control cells. In contrast, the WIN‐pocket mutant was expressed at a level comparable to WDR5‐WT but did not produce a comparable restoration of H3K4me3. These data indicate that WDR5 protein expression alone is insufficient and that an intact WIN pocket is required for efficient maintenance of global H3K4 trimethylation.

**FIGURE 3:**
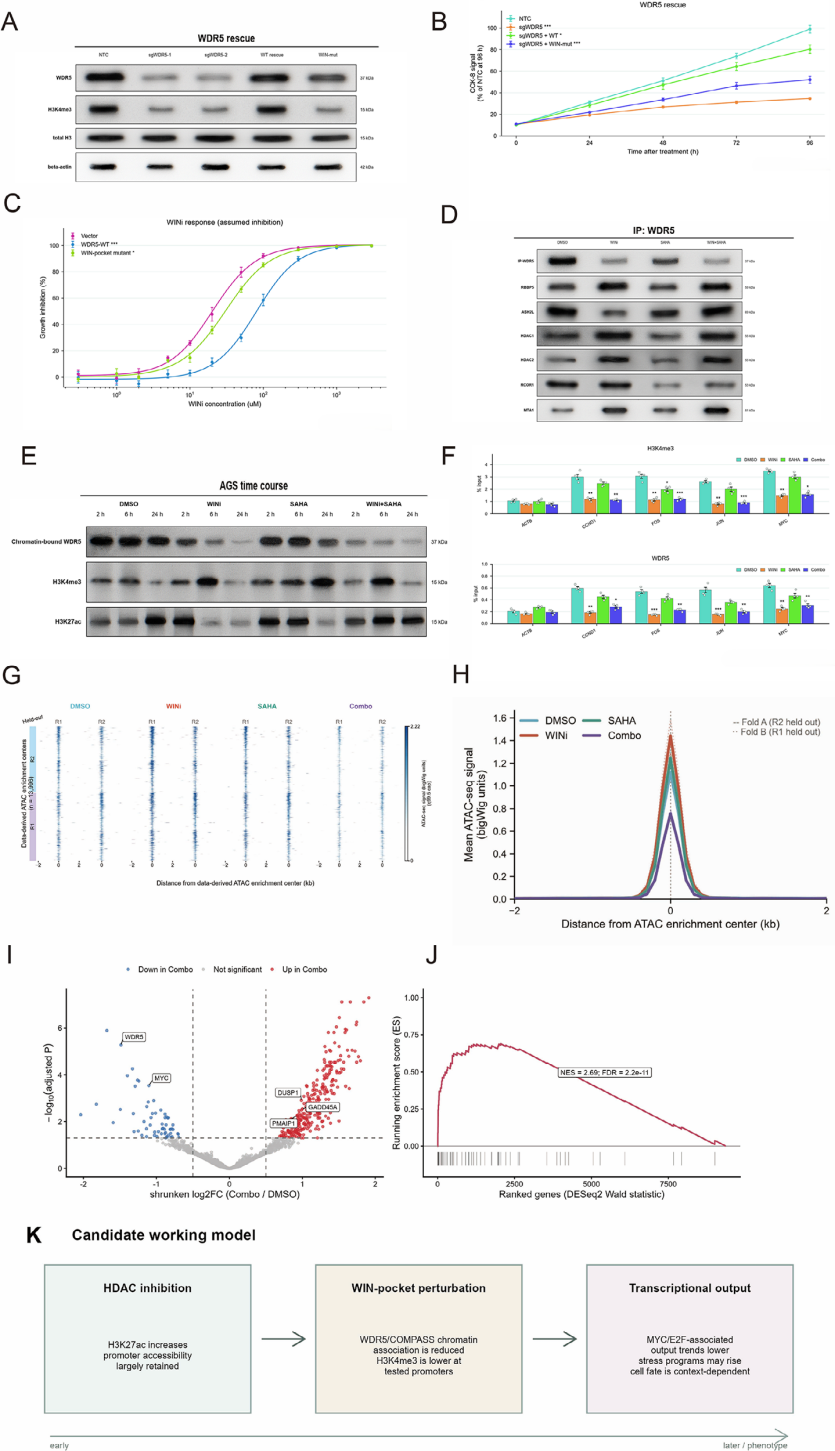
An intact WDR5 WIN pocket supports H3K4me3 maintenance and proliferative fitness and shapes the chromatin response to combined WIN and HDAC inhibition. (a) Immunoblot analysis following WDR5 depletion with two independent sgRNAs and rescue with sgRNA‐resistant WDR5‐WT or a WIN‐pocket‐defective WDR5 mutant. WDR5, H3K4me3, total histone H3, and β‐actin were examined in non‐targeting control cells, sgWDR5‐1 cells, sgWDR5‐2 cells, sgWDR5‐1 cells rescued with WDR5‐WT, and sgWDR5‐1 cells expressing the WIN‐pocket mutant. Total H3 and β‐actin are shown as reference controls for histone marks and whole‐cell protein abundance, respectively. (b) CCK8 time‐course proliferation curves in non‐targeting control cells, WDR5‐depleted cells, and WDR5‐depleted cells rescued with WDR5‐WT or the WIN‐pocket mutant. Relative CCK8 signal is plotted at 0, 24, 48, 72, and 96 h and normalized to the corresponding 96‐h control signal. (c) WINi dose–response curves for cells expressing vector, WDR5‐WT, or the WDR5 WIN‐pocket mutant. The displayed IC50 values are 20.6 µm for vector‐expressing cells, 73 µm for WDR5‐WT‐expressing cells, and 34.2 µm for cells expressing the WIN‐pocket mutant. (d) Representative WDR5 co‐immunoprecipitation analysis in AGS cells treated with DMSO, WINi, SAHA, or WINi plus SAHA. WDR5 immunoprecipitates were analyzed for WDR5, RBBP5, ASH2L, HDAC1, HDAC2, RCOR1, and MTA1. Because the amount of immunoprecipitated WDR5 differs among conditions, interaction patterns were interpreted qualitatively unless co‐precipitated proteins were normalized to the corresponding IP‐WDR5 signal. (e) Time‐course immunoblot analysis in AGS cells treated with DMSO, WINi, SAHA, or WINi plus SAHA for 2, 6, or 24 h. Chromatin‐bound WDR5, H3K4me3, and H3K27ac were examined. (f) WDR5 and H3K4me3 ChIP‐qPCR at the MYC, CCND1, FOS, JUN, and ACTB promoters following treatment with DMSO, WINi, SAHA, or WINi plus SAHA. WINi‐containing conditions reduce WDR5/H3K4me3 enrichment at growth‐associated promoters, whereas SAHA shows partial retention and ACTB remains comparatively stable. ChIP enrichment is expressed as a percentage of input. Bars show summary values, and individual measurements are overlaid. (g) Cross‐fitted, held‐out ATAC‐seq heatmaps centered on 13 996 data‐derived ATAC enrichment centers from −2 to +2 kb. R1 and R2 are displayed for DMSO‐, WINi‐, SAHA‐, and combination‐treated cells; the annotation at left indicates the held‐out replicate in each cross‐fitting fold. Signal is shown in bigWig units using the common q99.5‐capped scale displayed in the panel (0–2.22). Cross‐fitted mean ATAC‐seq profiles centered on the same data‐derived enrichment centers from −2 to +2 kb. Dashed traces denote Fold A (R2 held out), and dotted traces denote Fold B (R1 held out). The centered mean signal is highest under WINi, intermediate under DMSO and SAHA, and lowest under combined treatment; a distinct centered ATAC signal remains in all four conditions. (h) Cross‐fitted mean ATAC‐seq profiles over the same centers. (i) RNA‐seq volcano plot for the WINi plus SAHA vs. DMSO contrast. Blue points denote genes downregulated in the combination, pink points denote genes upregulated in the combination, and gray points denote non‐significant genes. Dashed lines indicate the displayed fold‐change and adjusted‐P‐value thresholds; selected genes are labeled. (j) Gene‐set enrichment analysis of the HALLMARK_MYC_TARGETS_V2 signature using the DESeq2 Wald‐statistic‐ranked RNA‐seq gene list. The displayed enrichment statistics are NES = 2.69 and FDR = 2.2 × 10^−11^. (k) Working model summarizing the proposed chromatin states. Baseline WDR5–COMPASS supports H3K4me3 and transcriptional competence. HDAC inhibition increases H3K27 acetylation, while ATAC‐enriched regions retain detectable accessibility with treatment‐dependent differences in signal amplitude. WIN‐pocket perturbation reduces WDR5/COMPASS chromatin association and lowers H3K4me3 at the tested promoters. Transcriptional output trends toward reduced MYC/E2F‐associated programs; stress programs may rise, and cell fate remains context‐dependent. The model is qualitative and does not imply identical changes at every promoter or unchanged promoter accessibility. Statistical significance is indicated as follows: ^*^ *p* < 0.05, ^**^ *p* < 0.01, ^***^ *p* < 0.001, and ^****^ *p* < 0.0001.

The chromatin phenotype was reflected in cellular proliferative fitness. In the CCK8 rescue time‐course assay, WDR5 depletion markedly reduced relative proliferation over 0–96 h, and this defect was substantially rescued by WDR5‐WT but not by the WIN‐pocket mutant (Figure 3b). Dose‐response analysis further showed that WDR5‐WT expression shifted the WINi response toward higher concentrations, with displayed IC50 values of 20.6 um in vector‐expressing cells, 73 um in WDR5‐WT‐expressing cells, and 34.2 um in cells expressing the WIN‐pocket mutant (Figure 3c). Thus, WDR5‐WT, but not the WIN‐pocket mutant, restored both CCK8‐measured proliferative capacity and relative resistance to WIN‐site inhibition.

We next examined how WINi and SAHA affected the WDR5‐associated protein complex. WDR5 immunoprecipitation revealed marked treatment‐dependent differences in both recovery of the WDR5 bait and co‐recovery of associated proteins (Figure 3d). The amount of immunoprecipitated WDR5 was strongly reduced following WINi treatment and was also lower in the combination condition than in DMSO‐ or SAHA‐treated cells. Despite the lower WDR5 bait recovery, RBBP5, HDAC1, HDAC2, and MTA1 remained prominent in WINi‐containing immunoprecipitates. ASH2L and RCOR1 exhibited distinct treatment‐dependent patterns, with ASH2L being relatively strong in the DMSO and combination conditions and RCOR1 being more evident in the DMSO and WINi conditions. Therefore, the raw co‐immunoprecipitation pattern is most consistent with non‐uniform reorganization of the WDR5 interactome rather than coordinated dissociation of all COMPASS and HDAC‐associated proteins.

Time‐course immunoblotting in AGS cells further revealed dynamic rather than strictly monotonic chromatin changes (Figure 3e). Chromatin‐bound WDR5 and H3K4me3 varied over the 2‐, 6‐, and 24‐h treatment intervals, without a uniform progressive decline under every condition. H3K27 acetylation was low in DMSO‐treated cells and increased in drug‐treated conditions. In the combination group, H3K27ac remained readily detectable at the later time points, whereas H3K4me3 was comparatively low at 24 h. The combination therefore produced a late state characterized by elevated H3K27ac together with reduced H3K4me3 in the representative experiment.

We next assessed WDR5 occupancy and H3K4me3 enrichment at selected promoters by ChIP–qPCR (Figure 3f). ChIP–qPCR showed high WDR5 and H3K4me3 enrichment under DMSO at the MYC, CCND1, FOS, and JUN promoters, partial retention under SAHA, and reduced enrichment in WINi‐containing conditions, with the combination among the lowest. This direction supports WIN‐pocket‐dependent loss of promoter licensing rather than drug‐induced promoter recruitment at the tested growth‐associated loci. ACTB remained comparatively stable and is best treated as a control locus rather than evidence for global promoter gain.

Genome‐wide ATAC‐seq profiling revealed condition‐dependent accessibility at data‐derived ATAC enrichment centers (Figure 3g,h). Cross‐fitted, held‐out heatmaps across 13 996 centers showed reproducible signal centered on these regions in both replicate folds. The corresponding mean profiles showed the highest centered ATAC‐seq signal after WINi treatment, intermediate signal under DMSO and SAHA, and the lowest signal under combined WINi plus SAHA treatment, although a distinct accessibility peak remained in all conditions.

RNA‐seq revealed substantial transcriptional remodeling under the differential‐expression contrast represented in the volcano plot, with 391 genes classified as upregulated and 39 classified as downregulated at the displayed thresholds (Figure 3i). Therefore, this panel shows a transcriptional response dominated numerically by gene induction rather than uniform genome‐wide repression. Gene‐set enrichment analysis identified a significant non‐random distribution of the HALLMARK_MYC_TARGETS_V2 signature across the DESeq2 Wald‐statistic‐ranked expression list, with the displayed statistics NES = 2.69 and FDR = 2.2 × 10^−11^ (Figure 3j).

### 2.3 Domain‐Resolved WDR5 Perturbation Identifies WIN‐ and WBM‐Associated Sensitization to SAHA and Convergent Chromatin Remodeling

To identify structural surfaces of WDR5 that modulate the response to HDAC inhibition, we performed domain‐focused CRISPR tiling across the WDR5 coding sequence in AGS and MKN45 cells under SAHA selection. Guide‐level effects were non‐uniformly distributed along WDR5, with several guides targeting the WIN site and the C‐terminal WBM‐associated region showing negative log2 fold changes under SAHA treatment (Figure 4a). After correction for the corresponding DMSO effects, negative SAHA‐specific ΔLFC values remained evident around the WIN and WBM regions in AGS cells, whereas the magnitude and regional separation of these effects were weaker in MKN45 cells (Figure 4b). These results nominate both surfaces as context‐dependent contributors to survival during SAHA exposure, although guides targeting other WDR5 surfaces also showed negative effects. These domain‐tiling data are interpreted as pooled guide‐depletion signals that nominate SAHA‐associated functional regions of WDR5, not as direct biochemical measurements of individual interface binding.

**FIGURE 4:**
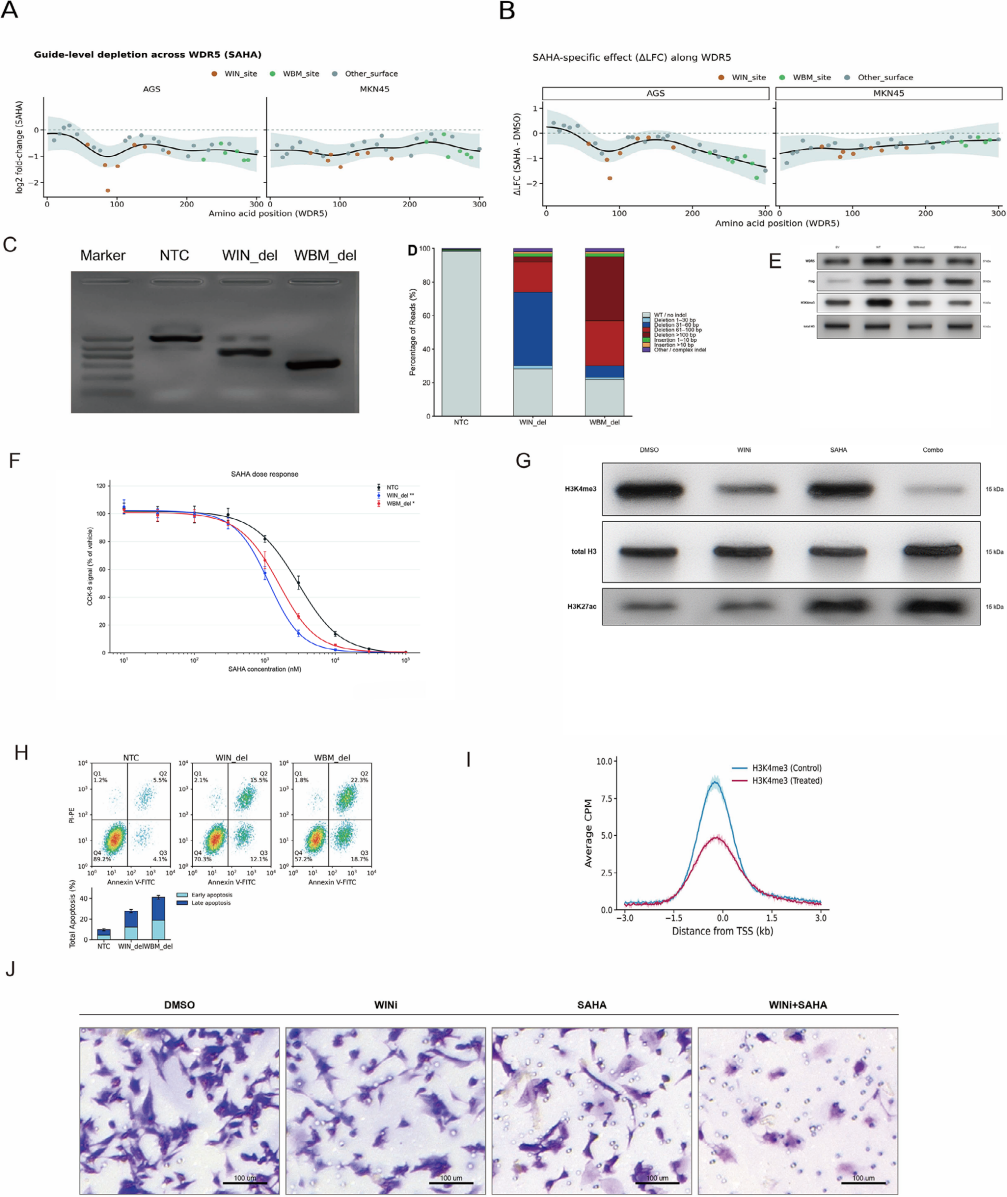
Domain‐resolved perturbation of WDR5 identifies WIN‐ and WBM‐associated sensitization to SAHA and convergent chromatin remodeling. (a) Guide‐level log2 fold changes under SAHA selection mapped to amino acid positions along WDR5 in AGS and MKN45 cells. Guides targeting the WIN site are shown in orange, guides targeting the WBM site in green and guides targeting other WDR5 surfaces in gray. Solid lines indicate locally smoothed trends, and shaded regions indicate the plotted uncertainty intervals. (b) SAHA‐specific guide effects along WDR5, calculated as ΔLFC = LFC(SAHA) − LFC(DMSO), in AGS and MKN45 cells. Negative ΔLFC values indicate preferential guide depletion under SAHA relative to DMSO. (c) PCR genotyping of non‐targeting control, WIN_del, and WBM_del cells. Shorter PCR products in the edited populations are consistent with enrichment of deletion‐containing alleles. The WIN_del and WBM_del lanes represent deletion‐enriched bulk‐like edited populations. (d) Amplicon‐sequencing analysis of editing outcomes in NTC, WIN_del, and WBM_del cells. Reads were classified into mutually exclusive categories: WT/no indel, deletion 1–30 bp, deletion 31–60 bp, deletion 61–100 bp, deletion >100 bp, insertion 1–10 bp, insertion >10 bp, and other/complex indel. WIN_del and WBM_del populations were enriched for deletion‐containing reads but retained residual WT/no‐indel reads. The retained WT/no‐indel fraction indicates heterogeneous edited populations rather than clonal isolates. (e) Immunoblot analysis of cells expressing empty vector, Flag‐tagged WDR5‐WT, WDR5 WIN mutant, or WDR5 WBM mutant. WDR5, Flag‐tag, H3K4me3, and total histone H3 were examined. Both mutant conditions showed lower H3K4me3 than WDR5‐WT. Because WDR5/Flag abundance was higher in WT than in mutant lanes, the apparent H3K4me3 reduction in mutants is interpreted with this expression difference in mind. (f) Fitted SAHA dose–response curves in NTC, WIN_del, and WBM_del cells. Normalized cell viability is plotted against log10‐transformed SAHA concentration. (g) Immunoblot analysis of H3K4me3, total histone H3, and H3K27ac following treatment with DMSO, WINi, SAHA, or WINi plus SAHA. (h) Representative Annexin V–FITC/PI flow‐cytometry profiles and summary of early‐ and late‐apoptotic fractions in NTC, WIN_del, and WBM_del cells. Q3 denotes Annexin V‐positive/PI‐negative early apoptosis, whereas Q2 denotes Annexin V‐positive/PI‐positive late apoptosis. (i) Average TSS‐centered H3K4me3 profiles for control cells and combination‐treated cells from −3 to +3 kb relative to transcription start sites. Signal is shown as average CPM. The treated trace denotes Combo (WINi plus SAHA). (j) Representative transwell migration images following treatment with DMSO, WINi, SAHA, or WINi plus SAHA. Scale bars, 100 µm. Statistical significance is indicated as follows: ^*^ *p* < 0.05, ^**^ *p* < 0.01, ^***^ *p* < 0.001, and ^****^ *p* < 0.0001.

To validate the domain‐level screen, we generated WIN‐region‐ and WBM‐region‐edited cell populations using paired CRISPR guides. PCR genotyping detected shorter amplicons in both WIN_del and WBM_del cells relative to the non‐targeting control, consistent with enrichment of deletion‐containing alleles (Figure 4c). Amplicon sequencing further showed that NTC cells were composed almost entirely of WT/no‐indel reads, whereas deletion‐containing reads predominated in both edited populations (Figure 4d). WIN_del cells were enriched primarily for intermediate‐sized deletions, particularly deletions of 31–60 bp, whereas WBM_del cells contained a larger contribution from 61–100‐bp and >100‐bp deletions. Approximately 20%–30% of reads in the edited populations remained WT/no‐indel, together with minor insertion and complex‐indel classes. Thus, these models represent deletion‐enriched, genetically heterogeneous populations rather than uniformly edited biallelic clones. Accordingly, WIN_del and WBM_del denote deletion‐enriched bulk‐like edited populations rather than single‐cell‐derived cloned lines.

We next examined whether the two WDR5 surfaces were required to maintain global H3K4 trimethylation. Flag immunoblotting confirmed detectable expression of WDR5‐WT, the WIN mutant, and the WBM mutant (Figure 4e). WDR5‐WT expression was associated with strong H3K4me3, comparable to that observed in the empty‐vector condition. In contrast, cells expressing either the WIN mutant or the WBM mutant displayed substantially lower H3K4me3 relative to total histone H3. Total WDR5 abundance was not fully matched among the three constructs, with the WT signal exceeding that of the mutants. Consistent with the domain‐tiling screen, both edited populations showed an apparent increase in SAHA sensitivity (Figure 4f). Because WT and mutant protein abundance was not matched, the lower H3K4me3 signal in the mutant lanes may reflect reduced mutant expression in addition to domain‐specific loss of function.

We then examined the histone‐modification state induced by pharmacological WIN‐site and HDAC inhibition. Endpoint immunoblotting showed that H3K4me3 was strongly reduced by WINi and was lowest in the WINi plus SAHA condition, whereas SAHA alone largely retained H3K4me3 at the bulk‐cell level (Figure 4g). Conversely, H3K27 acetylation was low under DMSO and WINi treatment but was markedly increased in SAHA‐containing conditions, with the strongest signal observed under combined treatment. Total histone H3 remained broadly stable. Thus, combined treatment generated a bulk chromatin state characterized by low H3K4me3 together with high H3K27ac.

Domain editing was also associated with increased apoptosis. Annexin V/PI analysis showed total Annexin V‐positive fractions of approximately 9.6% in NTC cells, 27.6% in WIN_del cells, and 41.0% in WBM_del cells, based on the combined early‐ and late‐apoptotic quadrants (Figure 4h). Both edited populations therefore displayed increased apoptosis‐associated fractions relative to NTC, with the largest increase observed in WBM_del cells under the conditions shown.

Genome‐wide H3K4me3 profiling revealed a lower average promoter‐proximal signal in the combination‐treated condition than in the corresponding control (Figure 4i). Both conditions retained a peak centered on transcription start sites, but the peak amplitude was substantially reduced following treatment. These data support broad attenuation, rather than complete elimination, of TSS‐proximal H3K4me3. Here, the treated condition denotes Combo (WINi plus SAHA).

Representative transwell migration assays showed fewer migrated cells after either WINi or SAHA treatment, with the lowest recovered‐cell density in the combined‐treatment condition (Figure 4j).

### 2.4 WIN–HDAC Co‐Targeting Attenuates H3K4me3 Enrichment and WDR5‐Associated Promoter Occupancy While Suppressing Nascent Growth‐Program Transcription

To determine whether the transcriptional effects of WIN–HDAC co‐targeting were accompanied by complete loss of accessible chromatin, we considered the cross‐fitted ATAC‐seq profiles shown in Figure 3g,h. All treatment groups retained a centered ATAC‐seq signal at data‐derived enrichment centers, although the average peak amplitude varied among conditions and was lowest after combined WINi plus SAHA treatment.

We next examined H3K4me3 signal using cross‐fitted and held‐out profiles centered on data‐derived H3K4me3 enrichment centers. The mean profile and heatmaps showed the strongest average centered signal under DMSO, followed by SAHA and WINi, with the lowest signal in the combination group across 9391 enrichment centers (Figure 5a,b). A representative interval at chr14:100 946 290–100 996 290 showed strong DMSO signal, intermediate monotherapy signals, and lower combination signal, with R1, R2, and condition‐mean traces displayed separately (Figure 5c). Because these panels are centered on data‐derived enrichment sites and the displayed interval is not assigned to a named gene, they support broad and representative H3K4me3 attenuation but do not independently establish TSS‐specific, promoter‐specific, or MYC‐locus changes.

**FIGURE 5:**
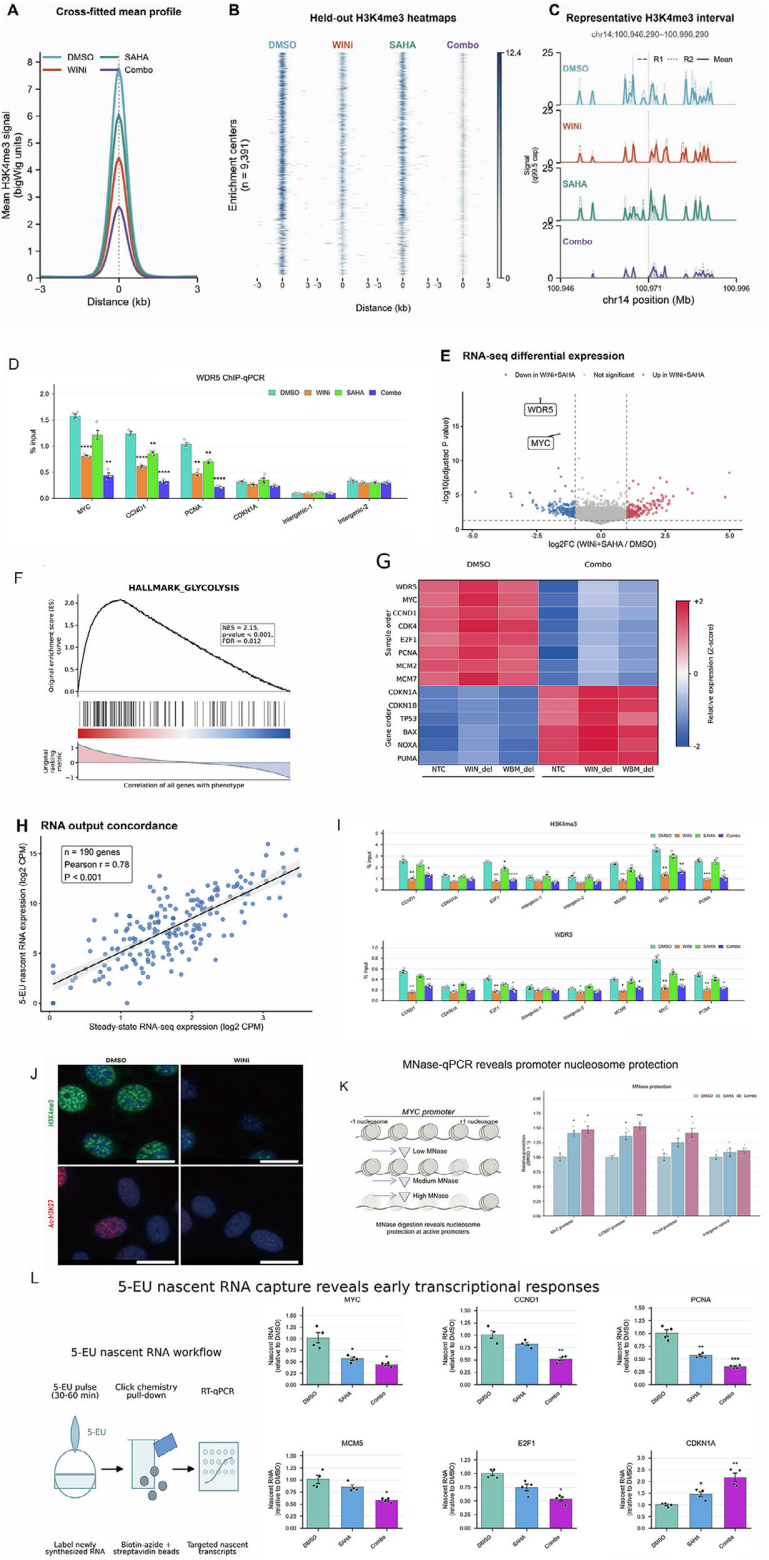
WIN–HDAC co‐targeting attenuates H3K4me3 enrichment and WDR5‐associated promoter occupancy while suppressing nascent growth‐program transcription. (a) Cross‐fitted mean H3K4me3 signal profiles centered on data‐derived H3K4me3 enrichment centers from −3 to +3 kb in DMSO‐, WINi‐, SAHA‐, and combination‐treated cells. Signal is shown in bigWig units. DMSO displays the highest centered mean signal, followed by SAHA and WINi, with the combination displaying the lowest signal. (b) Held‐out H3K4me3 heatmaps centered on the same 9391 data‐derived enrichment centers from −3 to +3 kb. DMSO, WINi, SAHA, and Combo are displayed using the common signal scale shown in the panel (0–12.4 bigWig units). (c) Representative H3K4me3 signal interval at chr14:100 946 290–100 996 290 under DMSO, WINi, SAHA, and combination treatment. R1 and R2 are shown as separate dashed/dotted traces together with the condition mean (solid trace) using a shared 0–25 signal scale. The panel is presented as a coordinate‐defined representative interval and is not assigned to a named gene. (d) WDR5 ChIP–qPCR at the MYC, CCND1, PCNA, and CDKN1A promoters and two intergenic control regions after DMSO, WINi, SAHA, or WINi plus SAHA treatment. Enrichment is expressed as a percentage of input; bars show mean ± s.e.m., and individual measurements are overlaid. WINi‐containing conditions reduce WDR5 enrichment at MYC, CCND1, and PCNA, whereas CDKN1A and the intergenic controls show lower signal and smaller differences. (e) RNA‐seq volcano plot for the WINi plus SAHA vs. DMSO contrast. Blue points denote genes downregulated by WINi plus SAHA, pink points denote upregulated genes, and gray points denote non‐significant genes. WDR5 and MYC are labeled among the downregulated genes. (f) Gene‐set enrichment analysis of the HALLMARK_GLYCOLYSIS signature using the ranked RNA‐seq gene list. The displayed statistics are NES = 2.15, nominal *p* < 0.001 and FDR = 0.012. (g) Heatmap of row‐scaled expression values for WDR5, proliferation‐associated genes, checkpoint genes, and apoptosis‐associated genes in NTC, WIN_del, and WBM_del cells treated with DMSO or WINi plus SAHA. (h) Correlation analysis between steady‐state RNA‐seq expression and 5‐EU nascent RNA expression across 190 genes. Axes show log2 CPM values. Pearson r = 0.78, *p* < 0.001. (i) WDR5 ChIP–qPCR and H3K4me3 ChIP–qPCR at the MYC, CCND1, PCNA, MCM5, E2F1, and CDKN1A promoters and two intergenic control regions after DMSO, WINi, SAHA, or WINi plus SAHA treatment. Enrichment is expressed as a percentage of input; bars show summary values with error bars and individual measurements overlaid. At the tested growth‐associated promoters, DMSO shows the highest enrichment, SAHA shows partial retention, and WINi‐containing conditions show lower WDR5 and H3K4me3 enrichment. (j) Representative immunofluorescence images of H3K4me3 and H3K27ac (labeled Ac‐H3K27 in the panel) in DMSO‐ and WINi‐treated cells. Green, H3K4me3; red, H3K27ac; blue, DAPI. Scale bars, as indicated. (k) MNase–qPCR analysis of promoter nucleosome protection. (l) 5‐EU nascent RNA capture assay. Statistical significance is indicated as follows: ^*^ *p* < 0.05, ^**^ *p* < 0.01, ^***^ *p* < 0.001, and ^****^ *p* < 0.0001.

Promoter‐level ChIP–qPCR supported reduced WDR5 occupancy at selected growth‐associated loci. At the MYC, CCND1, and PCNA promoters, WDR5 enrichment was lower after WINi treatment and lowest after combined WINi plus SAHA treatment, whereas the two intergenic controls remained low and changed little (Figure 5d). CDKN1A showed lower overall WDR5 enrichment and only modest variation among conditions.

RNA‐seq revealed broad transcriptional remodeling after treatment. In the WINi plus SAHA vs. DMSO contrast, the volcano plot labels WDR5 and MYC among downregulated genes, consistent with the expression heatmap and 5‐EU nascent RNA readouts (Figure 5e,g,l). Gene‐set enrichment analysis identified significant remodeling of the HALLMARK_GLYCOLYSIS program, with NES = 2.15, nominal *p* < 0.001, and FDR = 0.012 (Figure 5f). A curated expression heatmap further showed that combined treatment suppressed WDR5, MYC, CCND1, CDK4, E2F1, PCNA, MCM2, and MCM7, while increasing CDKN1A, CDKN1B, TP53, BAX, NOXA, and PUMA across NTC, WIN_del, and WBM_del backgrounds (Figure 5g). These data indicate a coordinated transcriptional switch from a proliferative MYC/E2F‐like state toward checkpoint and apoptotic programs. The correlation between steady‐state RNA‐seq and 5‐EU nascent RNA expression supports consistency between the analyzed expression readouts (Pearson r = 0.78, *p* < 0.001; Figure 5h).

We then measured WDR5 and H3K4me3 enrichment at an expanded set of selected promoters. At the MYC, CCND1, PCNA, MCM5, and E2F1 promoters, WDR5 ChIP–qPCR signal was highest under DMSO, partially retained under SAHA, and lower in WINi‐containing conditions (Figure 5i). H3K4me3 ChIP–qPCR showed the same overall ordering at these growth‐associated promoters. CDKN1A and the intergenic control regions had lower baseline WDR5 enrichment and smaller condition‐associated differences. These measurements support reduced WDR5/H3K4me3 enrichment at selected growth‐linked promoters after WIN‐site inhibition without establishing redistribution toward a checkpoint locus. Representative immunofluorescence images further showed lower global nuclear H3K4me3 staining after WINi, whereas H3K27ac (labeled Ac‐H3K27 in the panel) remained detectable (Figure 5j).

MNase‐qPCR was used as an orthogonal assay of promoter chromatin organization. SAHA and the combination increased MNase‐protected promoter DNA at MYC, CCND1, and PCNA, whereas the intergenic control remained largely unchanged (Figure 5k). This result indicates treatment‐dependent remodeling of nucleosome protection at growth‐associated promoters but is not interpreted as increased accessibility by itself.

Finally, 5‐EU nascent RNA capture demonstrated early transcriptional consequences of this chromatin state. Nascent MYC, CCND1, PCNA, MCM5, and E2F1 transcripts were reduced by SAHA and were further suppressed by WINi plus SAHA (Figure 5l). In contrast, nascent CDKN1A RNA increased under SAHA and reached the highest level under combined treatment. Together, these data show that WIN–HDAC co‐targeting reduces WDR5/H3K4me3 enrichment at selected growth‐associated promoters and rapidly suppresses nascent transcription of growth‐associated genes without completely eliminating signal at data‐derived ATAC‐enriched regions.

### 2.5 Combined WIN and HDAC Inhibition Suppresses Xenograft Growth with Concordant Epigenetic and Apoptotic Pharmacodynamics

To evaluate whether WIN‐HDAC co‐targeting suppresses tumor growth in vivo, we established luciferase‐positive subcutaneous gastric cancer xenografts in NOG mice and randomized animals into vehicle, SAHA, WINi, or combination treatment groups (Figure 6a). The schematic specifies randomization at approximately 100 mm3 or stable IVIS signal, treatment during Day 0–14 (or Day 21 where indicated), IVIS imaging at Day 0, Day 7, and Day 14, D‐luciferin at 150 mg/kg intraperitoneally with imaging at a fixed 10–15 min interval, and longitudinal caliper tumor‐volume plus body‐weight monitoring.

**FIGURE 6:**
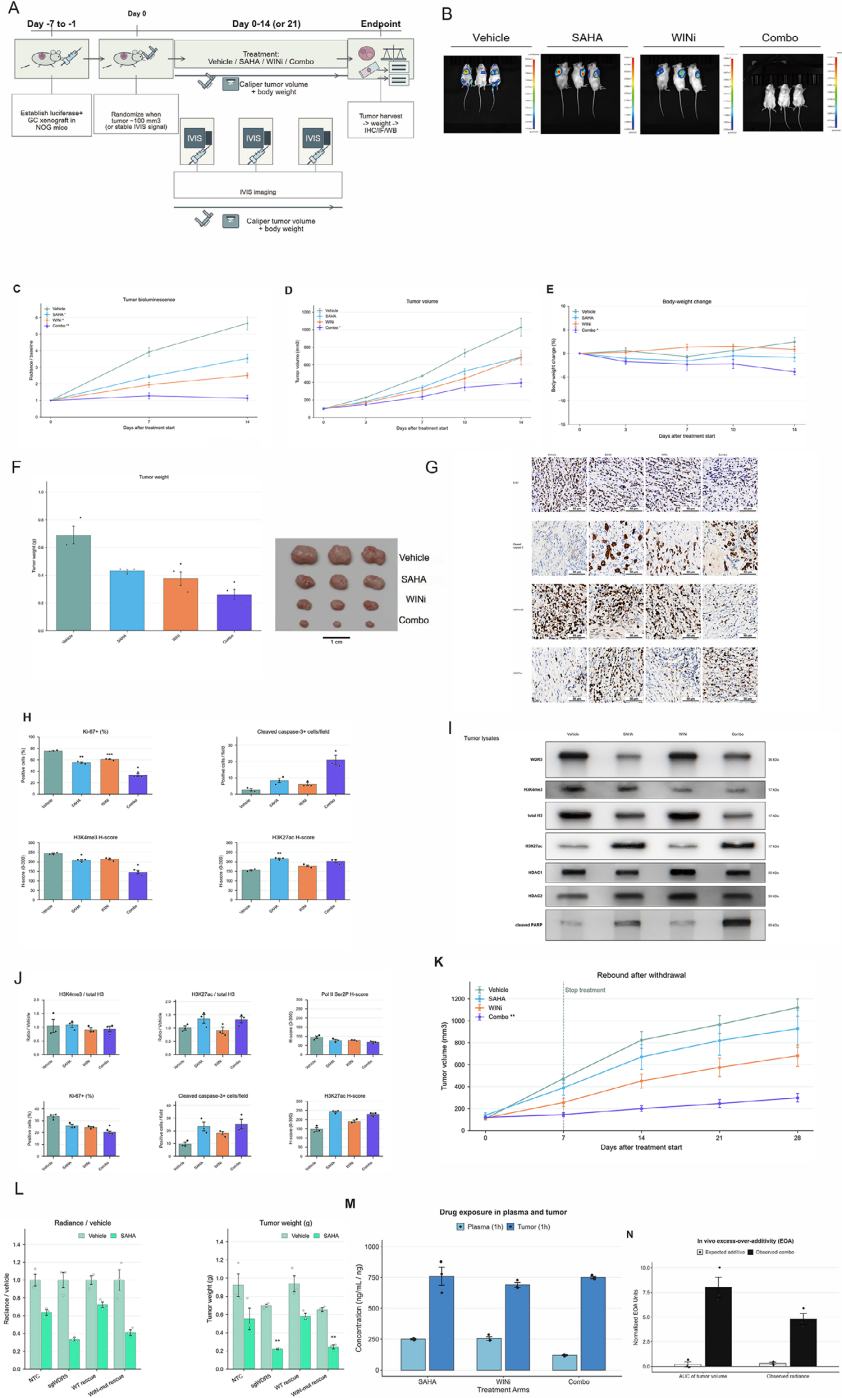
Combined WIN and HDAC inhibition suppresses subcutaneous luciferase‐positive gastric cancer xenograft growth with concordant epigenetic and apoptotic pharmacodynamics in NOG mice. (a) Schematic of the luciferase‐positive gastric cancer subcutaneous xenograft workflow in NOG mice. Mice bearing luciferase‐positive gastric cancer xenografts were randomized when tumors reached approximately 100 mm3 or stable IVIS signal, treated with vehicle, SAHA, WINi, or WINi plus SAHA, monitored by IVIS imaging at Day 0, Day 7, and Day 14 after D‐luciferin administration (150 mg/kg i.p., image at fixed 10–15 min), and followed by caliper tumor‐volume/body‐weight measurements and endpoint tissue collection. (b) Representative IVIS images from the indicated treatment groups. (c) Quantification of tumor bioluminescence over time, normalized to baseline signal. For the displayed time‐course data, *n* = 3 mice per group; longitudinal comparisons were analyzed using a mixed‐effects or repeated‐measures ANOVA model with multiple‐comparison correction. (d) Caliper‐based tumor‐growth curves for vehicle‐, SAHA‐, WINi‐, and WINi plus SAHA‐treated mice. For the displayed time‐course data, *n* = 3 mice per group; longitudinal comparisons were analyzed using a mixed‐effects or repeated‐measures ANOVA model with multiple‐comparison correction. (e) Body‐weight change over the treatment period as an initial tolerability readout. Body weight alone does not establish systemic safety and does not assess hematology, liver/kidney function, gastrointestinal toxicity, bone marrow or histology of normal proliferative tissues. (f) Endpoint tumor weight and representative images of excised tumors from each treatment group. (g) Representative immunohistochemistry images of Ki‐67, cleaved caspase‐3, H3K4me3, and H3K27ac in endpoint tumor sections from vehicle‐, SAHA‐, WINi‐ and combination‐treated mice. Scale bars, 50 µm. (h) Quantification of Ki‐67‐positive cells, cleaved caspase‐3‐positive cells per field, H3K4me3 H‐score and H3K27ac H‐score from endpoint tumor sections. (i) Immunoblot analysis of WDR5, H3K4me3, total histone H3, H3K27ac, HDAC1, HDAC2 and cleaved PARP in endpoint tumor lysates from the indicated treatment groups. (j) Early on‐treatment pharmacodynamic quantification. Top, H3K4me3 and H3K27ac immunoblot densitometry and Pol II Ser2P immunohistochemical H‐score. Bottom, Ki‐67‐positive cells, cleaved‐caspase‐3‐positive cells per field, and H3K27ac immunohistochemical H‐score in vehicle‐, SAHA‐, WINi‐, and combination‐treated tumors. (k) Tumor‐volume rebound after treatment withdrawal. The treatment‐on period (Day 0–7) and treatment‐off follow‐up period are indicated. For the displayed rebound analysis, *n* = 3 mice per group; longitudinal comparisons were analyzed using a mixed‐effects or repeated‐measures ANOVA model with multiple‐comparison correction. (l) In vivo WDR5‐dependency experiment. (m) Plasma and tumor drug‐exposure values measured 1 h after dosing in the labeled SAHA, WINi, and combination treatment arms. Bars show the displayed group summaries with individual measurements overlaid. (n) In vivo excess‐over‐additivity display comparing expected additive and observed combination values for tumor‐volume AUC and the radiance metric shown in the panel. Statistical significance is indicated as follows: ^*^ *p* < 0.05, ^**^ *p* < 0.01, ^***^ *p* < 0.001, and ^****^ *p* < 0.0001.

Representative IVIS images illustrate tumor‐associated bioluminescence in the four treatment groups at the displayed imaging time point (Figure 6b). Longitudinal quantification showed the greatest increase in normalized bioluminescence in vehicle‐treated mice, intermediate increases with either monotherapy, and the smallest increase with combined treatment (Figure 6c). Caliper‐based tumor‐volume measurements showed the same pattern: vehicle tumors grew most rapidly, SAHA and WINi produced intermediate tumor‐growth delay, and combined treatment produced the greatest suppression (Figure 6d). Body weight remained broadly stable across groups, suggesting no overt treatment‐associated weight loss during the observation window (Figure 6e).

Endpoint analysis confirmed the longitudinal imaging and volume data. Tumor weights were highest in the vehicle group, reduced by either SAHA or WINi and lowest in the combination group; excised tumor images visually reflected the same treatment ranking (Figure 6f). Immunohistochemical analysis showed that combined treatment was associated with reduced proliferation and increased apoptosis. Ki‐67 staining was highest in vehicle tumors, reduced in the monotherapy groups, and lowest in the combination group, whereas cleaved caspase‐3 staining showed the opposite trend and was highest after combined treatment (Figure 6g,h). H3K4me3 staining was reduced in WINi‐containing groups, while H3K27ac was increased in SAHA‐containing groups. The combination simultaneously showed low H3K4me3 and high H3K27ac, consistent with dual engagement of the WIN–H3K4me3 and HDAC–H3K27ac axes (Figure 6g,h).

Western blotting of endpoint tumor lysates provided an independent biochemical readout. H3K4me3 was reduced in WINi‐containing tumors, whereas H3K27ac was increased in SAHA‐containing tumors (Figure 6i). The combination group displayed low H3K4me3 together with high H3K27ac and showed the strongest cleaved PARP signal, consistent with enhanced apoptotic signaling. WDR5, HDAC1, and HDAC2 total protein levels did not show a uniform treatment‐dependent reduction, indicating that the most direct pharmacodynamic readouts are the histone‐mark changes and PARP cleavage.

Early on‐treatment pharmacodynamic profiling showed lower H3K4me3 abundance, Pol II Ser2P H‐score, and Ki‐67 positivity in combination‐treated tumors than in vehicle controls, together with higher H3K27ac abundance and H‐score and more cleaved‐caspase‐3‐positive cells (Figure 6j). After treatment withdrawal, tumors previously exposed to the combination exhibited the slowest post‐withdrawal growth, whereas vehicle and monotherapy groups grew more rapidly (Figure 6k). These data indicate that the combined treatment was associated with sustained suppression of tumor growth during the tested post‐treatment observation interval.

We next examined whether the in vivo phenotype depended on WDR5 functional status by comparing vehicle and SAHA responses in NTC, sgWDR5, WDR5‐WT‐rescue and WIN‐pocket‐mutant‐rescue xenografts (Figure 6l). Within the displayed within‐background normalization, SAHA‐treated radiance was lower in the sgWDR5 and WIN‐pocket‐mutant‐rescue groups than in the NTC and WDR5‐WT‐rescue groups. Endpoint tumor weight showed the same overall grouping: WDR5‐WT rescue restored an NTC‐like pattern, whereas the WIN‐pocket mutant remained closer to the sgWDR5 background. Drug‐exposure measurements in plasma and tumor 1 h after dosing are shown for the labeled treatment arms (Figure 6m). An excess‐over‐additivity display compares the expected additive and observed combination values for tumor‐volume AUC and the radiance metric shown in the panel (Figure 6n).

### 2.6 Pulse‐Washout and CCK8 Assays Reveal Sustained Impairment of Proliferative, Clonogenic and Biosynthetic Recovery within the Tested Washout Window

To test whether short‐term WIN‐HDAC co‐targeting produces sustained impairment of proliferative recovery within the tested washout window, we combined CCK8 time‐course readouts with a pulse‐washout clonogenic assay. For the clonogenic recovery experiment, cells were seeded at low density, treated with DMSO, WINi, SAHA, or WINi plus SAHA for 24, 48, or 72 h, washed extensively, and then cultured in drug‐free medium for long‐term colony formation (Figure 7a). This design follows the pulse‐washout clonogenic workflow described in the Methods and retains colony‐area quantification for Figure 7.

**FIGURE 7:**
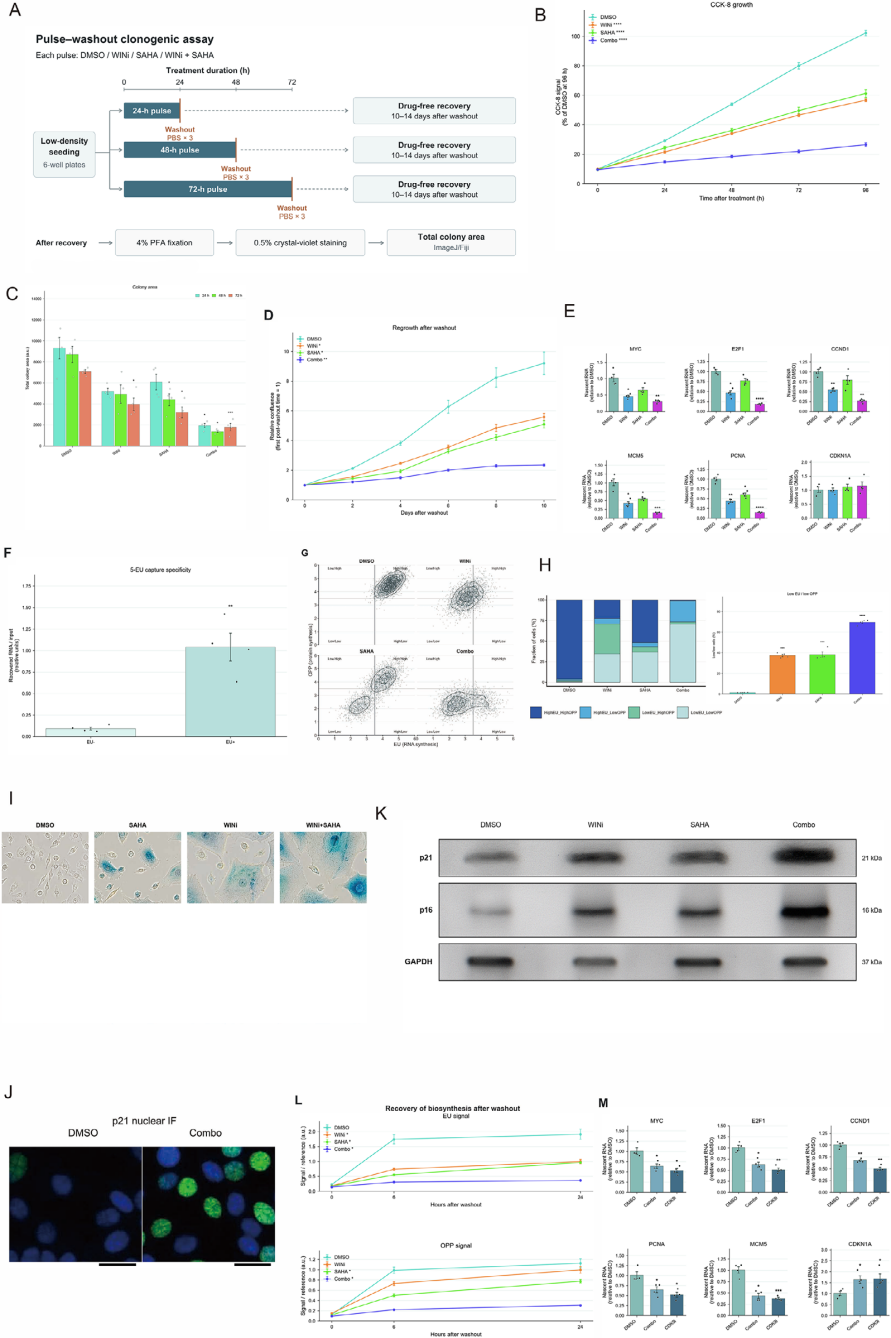
Pulse‐washout and CCK8 assays reveal sustained impairment of proliferative, clonogenic, and biosynthetic recovery within the tested washout window. (a) Schematic overview of the pulse–washout clonogenic workflow. Cells were seeded at low density, exposed to DMSO, WINi, SAHA, or WINi plus SAHA, washed three times with PBS, and then cultured in drug‐free medium before fixation and crystal violet staining. Treatment pulses of 24, 48, and 72 h were analyzed as quantified in c; the schematic is not drawn to scale. (b) CCK8 time‐course proliferation curve following treatment with DMSO, WINi, SAHA, or WINi plus SAHA. Relative CCK8 signal is plotted at 0, 24, 48, 72, and 96 h and normalized to the corresponding 96‐h control signal. (c) Quantification of total colony area after pulse–washout treatment. Bars indicate treatment durations of 24, 48, and 72 h. (d) Real‐time regrowth curves after drug washout, measured as cell confluence over time. (e) 5‐EU nascent RNA capture followed by RT–qPCR for MYC, E2F1, CCND1, MCM5, PCNA, and CDKN1A after treatment with DMSO, WINi, SAHA, or WINi plus SAHA. (f) 5‐EU labeling specificity control showing minimal pull‐down signal in EU‐negative samples and robust recovery in EU‐positive samples. (g) Representative EU/OPP flow‐cytometry plots measuring global RNA synthesis and protein synthesis after DMSO, WINi, SAHA, or WINi plus SAHA treatment. (h) Quantification of EU/OPP state distributions and the low‐EU/low‐OPP population fraction. (i) Representative SA‐β‐gal staining after DMSO, SAHA, WINi, or WINi plus SAHA treatment. (j) Representative p21 nuclear immunofluorescence images comparing DMSO and WINi plus SAHA treatment. (k) Immunoblot analysis of p21 and p16 after DMSO, WINi, SAHA, or WINi plus SAHA treatment. GAPDH was used as the loading control. (l) Recovery of global RNA and protein synthesis after drug washout, quantified as relative EU and OPP intensity at 0, 6, and 24 h after washout. (m) Relative nascent MYC, E2F1, CCND1, PCNA, MCM5, and CDKN1A RNA levels after DMSO, WINi plus SAHA (Combo), or CDK9‐inhibitor (CDK9i) treatment. Statistical significance is indicated as follows: ^*^ *p* < 0.05, ^**^ *p* < 0.01, ^***^ *p* < 0.001, and ^****^ *p* < 0.0001.

The CCK8 time‐course readout showed progressive expansion of DMSO‐treated cells, partial suppression by WINi or SAHA, and the lowest relative CCK8 signal in the WINi plus SAHA group over 0–96 h (Figure 7b). In the retained pulse‐washout clonogenic quantification, DMSO‐treated cells retained strong colony‐area recovery at all pulse durations, whereas SAHA and WINi monotherapies reduced total colony area and the combination produced the smallest residual colony area (Figure 7c). The reduction in colony area was generally greater after the longer treatment pulses, although the 48‐ and 72‐h combination values were similar rather than strictly monotonic. Real‐time regrowth analysis after washout showed that DMSO‐treated cells rapidly regained confluence, monotherapy‐treated cells showed partial recovery, and combination‐treated cells remained strongly growth‐impaired (Figure 7d). These results support sustained, duration‐dependent impairment of proliferative and clonogenic recovery during the tested washout interval after combined WIN and HDAC inhibition.

We next asked whether this sustained fitness defect was associated with early suppression of nascent transcription. 5‐EU capture showed that nascent MYC, E2F1, CCND1, MCM5, and PCNA transcripts were reduced by WINi or SAHA and were most strongly suppressed by the combination (Figure 7e). In contrast, nascent CDKN1A RNA increased, with the strongest induction in the combination group. EU‐negative controls showed minimal pull‐down signal, confirming the specificity of the 5‐EU labeling and capture workflow (Figure 7f).

To determine whether transcriptional suppression was coupled to reduced biosynthetic capacity, we performed dual EU/OPP labeling of RNA and protein synthesis. DMSO‐treated cells were enriched in the high‐EU/high‐OPP compartment, whereas WINi and SAHA shifted a fraction of cells toward reduced RNA and protein synthesis (Figure 7g,h). The combination produced the strongest population‐wide shift toward the low‐EU/low‐OPP state and generated the largest fraction of hypo‐biosynthetic cells (Figure 7h). This indicates coordinated suppression of both RNA and protein synthesis.

The long‐term recovery defect was accompanied by a senescence‐associated phenotype. SA‐β‐gal staining was low in DMSO‐treated cells, increased after either SAHA or WINi, and was strongest after combined treatment (Figure 7i). Nuclear p21 immunofluorescence was also increased after the combination compared with DMSO (Figure 7j). Immunoblotting confirmed induction of p21 and p16 by monotherapies and the strongest induction under combined treatment, with GAPDH remaining broadly stable (Figure 7k). These results support activation of a senescence‐like CDK‐inhibitory program after combined treatment.

Finally, we tested the recovery of biosynthetic capacity after drug removal. Following washout, EU and OPP intensities increased rapidly in the DMSO control and recovered partially after either monotherapy, whereas combination‐treated cells retained markedly lower EU and OPP intensities at 6 and 24 h (Figure 7l). Compared with DMSO, both WINi plus SAHA and the CDK9‐inhibitor comparator reduced nascent MYC, E2F1, CCND1, PCNA, and MCM5 RNA, whereas CDKN1A RNA was higher in both conditions (Figure 7m).

## 3 Discussion

This study advances a mechanistic model for why histone deacetylase (HDAC) inhibitors frequently show incomplete efficacy in solid tumors despite clear target engagement. The data support a principle that HDAC‐driven hyperacetylation is not, by itself, sufficient to extinguish oncogenic transcription when promoter licensing remains intact. Instead, promoter output becomes conditionally buffered by COMPASS‐mediated H3K4 methylation, and disabling this licensing layer through the WDR5 WIN pocket converts an “acetylated yet transcriptionally competent” promoter state into a less productive promoter state. This shifts the conceptual framework from viewing HDAC inhibitors as direct transcriptional suppressors to viewing them as agents that expose a dependency on orthogonal promoter‐competence modules—dependencies that can be therapeutically exploited when the appropriate licensing factor is co‐targeted [17, 18, 19, 20, 21, 22, 23, 24].

The integrated data support a mechanistic hierarchy across pharmacology, genetics, chromatin profiling, and nascent transcription readouts. The primary mechanism supported by multiple orthogonal assays is structural: WIN‐pocket perturbation reduces chromatin recovery of WDR5‐COMPASS‐associated components and attenuates promoter‐proximal H3K4me3 at growth‐linked loci. The causal link is strengthened by domain‐level CRISPR tiling and engineered WIN‐pocket‐defective rescue, which together place the WIN pocket upstream of the chromatin and fitness phenotypes. In parallel, SAHA rapidly increases H3K27 acetylation, confirming HDAC engagement. ATAC‐enriched regions retain a centered accessibility signal, but the mean amplitude is treatment‐dependent and lowest in the combination. The combined perturbation therefore creates a hyperacetylated state with attenuated H3K4me3‐based promoter competence and reduced average ATAC signal at data‐derived accessible regions, under which MYC/E2F transcriptional programs are strongly suppressed. Secondary consequences include cell‐cycle arrest, apoptosis and/or senescence‐like features, reduction in migration‐ and invasion‐associated readouts, and sustained impairment of clonogenic recovery during the tested washout interval.

The strongest causal evidence places the WDR5 WIN pocket upstream of global H3K4me3 maintenance and proliferative fitness, whereas the sufficiency of promoter H3K4me3 loss for transcriptional suppression remains to be directly tested. Genetic WDR5 loss reduces global H3K4me3 and fitness, and WDR5‐WT–but not WIN‐pocket‐defective variants–restores these outputs, supporting a functional requirement for the WIN pocket in these contexts. Pharmacologic WIN‐site inhibition phenocopies these effects and shows diminished potency in WIN‐pocket mutant backgrounds, supporting on‐target engagement. ChIP‐level evidence links reduced WDR5 occupancy and diminished H3K4me3 at the same promoters, and genome‐wide H3K4me3 profiling extends this attenuation to data‐derived H3K4me3 enrichment centers. The exact molecular step linking H3K4me3 attenuation to reduced transcriptional output and impaired fitness remains unresolved: possibilities include altered Pol II recruitment, promoter escape, elongation, or pause‐release dynamics. The similarity between the combination's nascent transcription suppression and a CDK9 inhibitor signature is consistent with an elongation‐associated impairment, but this remains an inference without direct Pol II occupancy and phosphorylation‐state mapping [25, 26].

These findings integrate with, and extend, existing literature in three ways. First, they build on structural and chemical biology work establishing the WDR5 WIN pocket as a tractable interface required for MLL/SET1 complex assembly and H3K4 methylation output [6, 7, 8, 9, 18]. Second, they align with studies linking WDR5 to MYC‐driven transcriptional amplification and promoter engagement, positioning WDR5 not only as a COMPASS scaffold but also as a node that stabilizes high‐output growth programs [10, 11, 27]. Third, they speak to a broader theme in epigenetic therapy: that targeting one chromatin layer can unmask buffering by another, producing context‐dependent vulnerabilities that are not apparent under baseline conditions [4, 5, 17, 18, 19, 20, 21]. In this framing, HDAC inhibition acts as a stressor that increases acetylation while accessibility remains detectable at data‐derived ATAC‐enriched regions; WDR5–COMPASS provides the counterweight preserving promoter competence, and co‐targeting removes this support and drives coordinated program shutdown.

A notable aspect of the current data is that transcriptional suppression does not require complete loss of accessible chromatin. ATAC‐seq profiles centered on data‐derived accessible regions showed a sharp centered signal in every condition, but the mean amplitude varied and was lowest after combined treatment. Because these regions were not anchored to transcription‐start‐site annotations in the updated analysis, the data do not establish that promoter accessibility is unchanged. Together with reduced H3K4me3 at data‐derived enrichment centers and reduced WDR5/H3K4me3 ChIP–qPCR signal at selected growth‐associated promoters, the findings support transcriptional suppression without wholesale chromatin closure, while leaving the promoter‐specific relationship between accessibility and licensing to be tested directly. This distinction may help explain why HDAC inhibition can alter acetylation without consistently extinguishing tumor‐supporting transcriptional programs.

The work also delineates heterogeneity and a plausible biomarker axis. Baseline WDR5 abundance and H3K4me3 levels correlate with combination sensitivity, and diffuse/SRCC models appear enriched for vulnerability. While these associations support a “dependency‐by‐abundance” stratification framework, the causal direction is not fully resolved. High WDR5 may mark tumors with elevated transcriptional demand and MYC/E2F reliance, or may correlate with a chromatin state that is more COMPASS‐dependent for promoter maintenance under stress. Distinguishing “driver” from “marker” will require perturbation studies that titrate WDR5 abundance and test whether altering baseline WDR5 is sufficient to shift combination response independently of subtype background.

Several limitations were considered. First, the mechanistic step linking H3K4me3 erosion to transcriptional suppression is inferred from nascent RNA readouts and pathway signatures rather than directly mapped with Pol II ChIP‐seq/CUT&RUN, GRO‐seq/PRO‐seq, or phosphorylation‐state profiling of Pol II (Ser2/Ser5) and elongation factors. This limits precision in assigning the primary transcriptional defect (initiation vs. pause release vs. elongation). Second, while xenograft data support in vivo activity and mechanism‐consistent pharmacodynamics, the models are NOG mouse subcutaneous xenografts and may not capture gastric cancer heterogeneity, orthotopic tumor architecture, stromal interactions, immune‐mediated consequences of HDAC inhibition or WDR5 targeting, or clinical tolerability boundaries. In addition, the current safety assessment is limited to body‐weight monitoring and gross observation; it does not include hematology, liver and kidney function, gastrointestinal toxicity, bone‐marrow assessment, or histology of normal proliferative tissues. Third, WIN‐site inhibitors and SAHA are used as representative chemical tools; class‐wide generality (across multiple WIN antagonists and HDAC1/2‐selective inhibitors vs. pan‐HDAC agents) is suggested but not fully established in the presented scope. Fourth, resistance mechanisms are not yet defined: tumors and cell lines may adapt by rewiring H3K4 methylation via alternative complexes, altering acetylation dynamics, or reducing reliance on MYC/E2F programs.

Targeted future experiments would directly address these gaps. (1) Map Pol II distribution and dynamics under SAHA, WINi, and combination using Pol II CUT&RUN/ChIP‐seq (total and Ser2P/Ser5P) and quantify pausing indices at growth‐linked promoters; complement with PRO‐seq to measure transcriptional engagement genome‐wide. (2) Test whether restoring H3K4me3 independently of WDR5‐COMPASS rescues transcription and fitness under combination, to distinguish whether H3K4me3 loss is necessary and sufficient for the impaired promoter‐output state. (3) Define the role of MYC dependence explicitly by perturbing MYC (knockdown or degron) and assessing epistasis with combined WINi and SAHA treatment; evaluate whether MYC‐high states are associated with deeper synergy. (4) Identify acquired resistance by long‐term selection under combination, followed by whole‐exome sequencing and chromatin profiling to uncover compensatory pathways. (5) Extend in vivo testing to patient‐derived xenografts, orthotopic gastric cancer models, and, where feasible, immunocompetent or humanized models to assess immune and stromal contributions. (6) Perform formal tolerability studies using clinically relevant schedules, including complete blood counts, serum chemistry for liver and kidney function, gastrointestinal assessment, bone‐marrow evaluation, and histology of normal proliferative tissues such as intestinal crypts and hematopoietic compartments.

From a translational perspective, the data support a plausible application boundary: tumors with high WDR5 and/or high promoter H3K4me3–and particularly those with diffuse/SRCC features–may be more likely to benefit from WIN‐pocket blockade combined with HDAC inhibition. However, several evidentiary gaps remain before clinical positioning is justified. First, safety and therapeutic index need formal evaluation, especially because WDR5 and H3K4 methylation contribute to normal proliferative tissues. Body weight stability in the current xenograft experiments is therefore viewed only as an initial tolerability observation, not as evidence of systemic safety. Second, pharmacokinetic and pharmacodynamic relationships remain to be defined for clinically viable WIN antagonists and for HDAC inhibitors with improved selectivity, including tumor penetration and schedule optimization. Third, candidate response‐associated biomarker features require operationalization: quantification of WDR5 and H3K4me3 by IHC or proteomics requires standardization and validation in independent cohorts, ideally linked to ex vivo drug response assays. Finally, combination benefit requires benchmarking against current standards‐of‐care and rational partners in models that capture clinical heterogeneity.

## 4 Method

### 4.1 Cell Culture

Human gastric cancer cell lines (including AGS and MKN45) were obtained from authenticated sources and were cultured at 37 °C in a humidified incubator with 5% CO_2_. AGS cells were maintained in F‐12K medium; MKN45 cells were maintained in RPMI‐1640. All media were supplemented with 10% fetal bovine serum (FBS) and 1% penicillin–streptomycin. For luciferase xenograft studies, AGS and/or MKN45 were transduced with a lentiviral luciferase construct.

### 4.2 Drug Treatments

Vorinostat (SAHA) and WDR5 WIN‐site inhibitor (WINi) were prepared as concentrated stocks in DMSO and stored at −20 °C in aliquots protected from light. Working solutions were freshly prepared in complete medium. DMSO concentration was held constant across conditions (final DMSO ≤0.1% v/v).

### 4.3 Lentiviral Production and Transduction

Lentivirus was produced in HEK293T cells by co‐transfection of transfer plasmids with packaging plasmids (psPAX2 and pMD2.G) using a lipid‐based transfection reagent. Viral supernatants were harvested at 48 and 72 h, filtered through 0.45 µm filters, and used immediately or stored at −80 °C. Target cells were transduced in the presence of polybrene and selected with the corresponding antibiotic until non‐transduced controls were eliminated. For rescue experiments, cells were transduced with sgRNA‐resistant WDR5 constructs and selected prior to phenotypic assays.

### 4.4 Epigenetic‐Focused Pooled CRISPR Knockout Screening

Epigenetic‐focused pooled CRISPR‐Cas9 knockout screens were performed in AGS and MKN45 cells stably expressing Cas9. Cells were transduced with the epigenetic‐focused sgRNA library used for the screen. This library contained 24 615 sgRNAs per comparison, targeting 758 epigenetic/chromatin‐regulatory genes with 30 sgRNAs per regular target gene, plus safe‐targeting control guides (AAVS1 = 1671; CCR5 = 78; ROSA26 = 126). Cells were infected at a multiplicity of infection (MOI) of 0.2–0.3 to favor single sgRNA integration, selected with puromycin for 5–7 days, and maintained with sufficient coverage to preserve library representation.

Following selection, baseline samples (T0) were collected for genomic DNA (gDNA). Cells were then split into DMSO and SAHA treatment arms at equal representation and cultured under drug selection pressure for the specified duration, 14 days for the main screen, while maintaining guide representation at each passage. gDNA was extracted using a column‐ or precipitation‐based method. sgRNA cassettes were PCR‐amplified using a two‐step PCR protocol to append Illumina adapters and indices, with multiple parallel PCR reactions per sample to avoid saturation. Libraries were sequenced on an Illumina platform, and sgRNA abundance was quantified against the epigenetic‐focused reference library.

Normalized sgRNA counts were used to calculate SAHA‐vs.‐DMSO log2 fold changes and guide‐level depletion/enrichment statistics. Gene‐level screen summaries were generated by aggregating guide‐level effects for each target gene and applying multiple‐testing correction. Candidate sensitization genes were defined as targets whose guides were depleted under SAHA relative to DMSO, whereas candidate resistance genes were defined as targets whose guides were enriched under SAHA.

### 4.5 Domain‐Tiling CRISPR Mutagenesis of WDR5

A domain‐tiling sgRNA library targeting the WDR5 coding region was designed to densely cover exons across the WDR5 locus, including guides spanning the WIN pocket and WBM interface. Non‐targeting controls and essential‐gene controls were included. Lentiviral delivery, selection, and coverage criteria followed the epigenetic‐focused pooled‐screen workflow described above.

After puromycin selection, cells were exposed to DMSO or SAHA for the defined period while maintaining guide representation. gDNA was collected at baseline and endpoint, and sgRNA abundance was quantified by deep sequencing. Guide‐level effects were mapped to amino acid positions, and domain‐specific sensitization was inferred by comparing SAHA vs DMSO depletion after subtracting baseline fitness penalties.

### 4.6 Generation of WDR5 Domain‐Deletion and Mutant Rescue Cell Lines

WIN_del and WBM_del alleles were generated using CRISPR–Cas9 with paired sgRNAs flanking the targeted region. Edited cells were maintained and analyzed as deletion‐enriched bulk‐like populations after selection and genotype enrichment. Genotypes were confirmed by PCR across the edited locus followed by Sanger sequencing and/or amplicon deep sequencing.

For rescue experiments, sgRNA‐resistant WDR5 (WT) and WIN‐pocket mutant constructs were expressed from lentiviral vectors. Expression was confirmed by immunoblotting. On‐target engagement by WINi was assessed by comparing drug‐response shifts between WT‐rescued and mutant‐rescued lines using matched dosing schedules.

### 4.7 Cell Viability, CCK8 Time‐Course Proliferation Assays, Growth‐Rate (GR) Metrics, and Drug Synergy

Dose‐response assays were performed in 96‐ or 384‐well plates seeded at densities optimized for logarithmic growth over the assay window. Cells were treated with single agents or combinations using a matrix design. Endpoint viability was measured using a luminescence‐based CCK8. Time‐zero controls were included to compute GR values. GR metrics were calculated as described by Hafner et al., using cell counts or proxy viability signals at time zero and endpoint, and were implemented in R/Python with documented code. For CCK8 time‐course proliferation assays, cells were seeded in 96‐well plates at matched starting densities and treated with DMSO, WINi, SAHA, WINi plus SAHA, or the indicated genetic‐rescue conditions. At 0, 24, 48, 72, and 96 h, CCK8 reagent was added according to the manufacturer's protocol, and absorbance was measured at 450 nm after incubation. Blank‐medium readings were subtracted before analysis.

Synergy was quantified using Bliss independence and ZIP models (23,24). For ZIP scores, the synergyfinder package or equivalent was used with default settings unless specified. For Bliss, expected combination effects were calculated from single‐agent response surfaces, and excess inhibition was computed per dose pair. Multiple‐testing correction was applied where appropriate.

### 4.8 Pulse‐Washout Clonogenic Assays

For the pulse‐washout clonogenic assays shown in Figure 7, cells were seeded at low density in 6‐well plates. Cells were treated with DMSO, WINi, SAHA, or a combination for 24, 48, or 72 h. Drugs were removed by aspirating media and washing three times with PBS, followed by incubation in drug‐free medium for 10–14 days with media replacement every 3–4 days. Colonies were fixed with 4% paraformaldehyde and stained with 0.5% crystal violet. Plates were imaged using a flatbed scanner or imaging system. Total colony area was quantified using ImageJ/Fiji with fixed thresholding parameters per experiment.

For regrowth after washout, cells were monitored in real time using an IncuCyte system or equivalent. Confluence was measured at fixed intervals for up to 10 days post‐washout. Confluence curves were normalized to the first post‐washout timepoint.

### 4.9 Migration and Invasion Assays

For transwell migration assays, cells were serum‐starved as specified, then seeded into transwell inserts in serum‐free medium. Complete medium containing chemoattractant was placed in the lower chamber. After incubation for 24 h, inserts were fixed and stained with crystal violet. Non‐migrated cells were removed from the upper surface using cotton swabs, and migrated cells were imaged in multiple random fields per insert.

For Matrigel invasion, inserts were coated with growth factor‐reduced Matrigel at the specified dilution and polymerized before cell seeding. Invasion proceeded for 24–48 h as optimized per line.

### 4.10 EdU Incorporation and Apoptosis Assays

EdU incorporation was performed using a click‐chemistry EdU kit. Cells were pulsed with EdU for 30–60 min, fixed, and processed per manufacturer instructions. Nuclei were counterstained with DAPI, and EdU‐positive cells were imaged by fluorescence microscopy using matched exposure settings across conditions.

Apoptosis was measured using Annexin V/propidium iodide (PI) staining. Cells were harvested, washed with binding buffer, stained with Annexin V and PI, and analyzed by flow cytometry within 1 h. Early and late apoptotic fractions were defined by standard quadrant gating.

### 4.11 Senescence Assays

Senescence‐associated β‐galactosidase staining was performed using standard X‐gal protocol. Cells were fixed and incubated with SA‐β‐gal staining solution at 37 °C (no CO_2_) until blue signal developed for 16 h. Images were acquired using a brightfield microscope.

### 4.12 EU Nascent RNA Capture and RT–qPCR

Nascent transcription was quantified by 5‐ethynyl uridine (5‐EU) metabolic labeling. Cells were treated with DMSO, WINi, SAHA, or a combination for the specified duration, pulsed with 5‐EU, and harvested. Total RNA was extracted using TRIzol with on‐column DNase treatment. 5‐EU‐labeled RNA was biotinylated using copper‐catalyzed click chemistry and captured on streptavidin beads. EU− controls (no 5‐EU) were processed in parallel to assess background binding. Captured RNA was eluted and reverse‐transcribed using random hexamers. Target transcripts were quantified by qPCR using SYBR Green. Values were normalized to internal controls as specified and expressed relative to DMSO. For the CDK9‐inhibitor comparator shown in Figure 7m, inhibitor‐treated cells were processed in parallel with DMSO‐ and WINi‐plus‐SAHA‐treated cells through 5‐EU labeling, capture, and RT–qPCR, and target‐transcript values were expressed relative to DMSO.

### 4.13 EU/OPP Dual Labeling of RNA and Protein Synthesis

Global RNA synthesis was measured by EU incorporation, and protein synthesis was measured by O‐propargyl puromycin (OPP) labeling using click‐chemistry detection kits. Cells were treated as indicated, pulsed with EU and OPP sequentially or concurrently, fixed, and conjugated to fluorophores by click chemistry. Samples were analyzed by flow cytometry. Quadrant thresholds were set based on DMSO controls and single‐label controls. State distributions were reported as fractions of total singlets after debris and doublet exclusion.

For washout recovery assays, EU/OPP labeling was performed at 0, 6, and 24 h following washout and quantified as above.

### 4.14 RNA‐Seq and 5‐EU‐Based Nascent‐Transcription Analysis

For steady‐state RNA‐seq, total RNA was extracted from treated cells with DNase treatment. RNA integrity was assessed. Poly(A)+ selection was performed prior to library construction. Libraries were sequenced on an Illumina platform to obtain paired‐end reads.

RNA‐seq reads were aligned to the human genome (GRCh38) using STAR, and gene‐level counts were generated using featureCounts. Differential expression was performed using DESeq2, with FDR control as defined in ‘Statistical analysis.’ GSEA was performed using MSigDB Hallmark gene sets and fgsea, with gene ranking by signed statistic. The nascent‐transcription panels in the current figure set were generated from 5‐EU capture.

### 4.15 ATAC‐seq

ATAC‐seq was performed as described with minor modifications. Briefly, 50 000 viable cells per condition were harvested, washed in cold PBS, and lysed to isolate nuclei. Transposition was performed using Tn5 transposase at 37 °C for 30 min. DNA was purified, and libraries were PCR‐amplified with indexed primers; amplification cycles were determined by qPCR to avoid saturation. Libraries were size‐selected and sequenced paired‐end.

Reads were trimmed, aligned to GRCh38 using Bowtie2, and duplicates were removed. Peaks were called using MACS2 with ATAC‐specific parameters. TSS‐centered analyses, where used, were generated with GENCODE annotations. For Figure 3g,h, replicate‐level ATAC‐seq signal was evaluated using reciprocal cross‐fitting: data‐derived ATAC enrichment centers were defined in one fold and signal was displayed in the held‐out replicate, with R1 and R2 alternately held out. Heatmaps and mean profiles were centered on 13 996 retained centers over ±2 kb. Signal was displayed in bigWig units, with the heatmap scale capped at the q99.5 value shown in the panel.

### 4.16 ChIP–qPCR

Chromatin immunoprecipitation (ChIP) was performed using formaldehyde crosslinking (final 1% for 10 min), quenched with glycine. Cells were lysed, and chromatin was sheared by sonication to ∼200–500 bp. Equivalent chromatin input was incubated overnight with antibodies against WDR5 and H3K4me3, followed by capture with protein A/G beads. After sequential washes, DNA was eluted, reverse‐crosslinked, and purified. qPCR was performed using primers targeting growth‐associated promoters and control loci, including MYC, CCND1, FOS, JUN, PCNA, E2F1, MCM‐family genes, CDKN1A, ACTB, and intergenic controls as appropriate for each panel. ChIP enrichment was expressed as % input. Each ChIP included at least two technical qPCR replicates per primer pair.

### 4.17 CUT & Tag

CUT&Tag was performed using a commercial kit [13]. Briefly, 50 000–2 00 000 cells per sample were immobilized on concanavalin A beads, permeabilized, and incubated with the primary antibody followed by a secondary antibody where required. pA‐Tn5 was added, and tagmentation was initiated under controlled conditions. DNA fragments were purified, PCR‐amplified with indexed primers, and sequenced.

Reads were aligned to GRCh38, filtered for mapping quality, and duplicate reads were removed. Signal tracks were represented as normalized bigWigs. TSS‐centered H3K4me3 analyses, including Figure 4i, were computed relative to annotated transcription start sites. For Figure 5a,b, reciprocal held‐out profiles were centered on 9391 data‐derived H3K4me3 enrichment centers over ±3 kb rather than on annotated TSSs. Figure 5c displays R1, R2, and condition‐mean H3K4me3 signal across chr14:100 946 290–100 996 290 using a shared *y*‐axis.

### 4.18 MNase–qPCR

MNase digestion was used to assess promoter nucleosome protection. Nuclei were isolated from treated cells and aliquoted equally. MNase digestion was performed at low/medium/high enzyme concentrations at 37 °C and terminated with EGTA. DNA was purified and quantified. qPCR was performed on promoter regions and an intergenic control using primer pairs designed to report nucleosome‐protected fragments. Protection was calculated as the relative abundance of target amplicons in digested vs. input DNA, normalized across conditions.

### 4.19 Subcellular Fractionation and Chromatin Extraction

Cells were harvested on ice and fractionated into cytosolic, nuclear, and chromatin‐bound fractions using a detergent‐based sequential extraction protocol. Briefly, cytosolic proteins were extracted in hypotonic buffer containing NP‐40 and protease inhibitors, nuclei were pelleted, and soluble nuclear proteins were extracted with moderate salt. Chromatin‐bound proteins were extracted by nuclease digestion. Fraction purity was validated by immunoblotting for Tubulin (cytosol), Lamin B1 (nucleus), and Histone H3 (chromatin). WDR5, RBBP5, HDAC1, and HDAC2 distributions were assessed by western blot.

### 4.20 Immunoblotting

Cells or tumor tissues were lysed in RIPA buffer supplemented with protease and phosphatase inhibitors. Protein concentration was determined by BCA assay. Equal protein amounts were resolved by SDS–PAGE and transferred to PVDF membranes. Membranes were blocked in 5% milk and incubated with primary antibodies followed by HRP‐conjugated secondary antibodies. Signal was detected by chemiluminescence and quantified by densitometry in ImageJ. For histone marks, signals were normalized to total H3.

### 4.21 Immunofluorescence and Immunohistochemistry

For immunofluorescence, cells on coverslips were fixed in 4% paraformaldehyde, permeabilized in 0.1%–0.3% Triton X‐100, blocked in serum‐containing buffer, and incubated with primary antibodies followed by fluorophore‐conjugated secondary antibodies. Nuclei were counterstained with DAPI. Images were acquired on a fluorescence microscope using identical exposure settings across conditions within experiments.

For xenograft tumors, formalin‐fixed paraffin‐embedded (FFPE) sections were deparaffinized, subjected to antigen retrieval, blocked, and stained for Ki‐67, cleaved caspase‐3, H3K4me3, and H3K27ac. Signal was developed with DAB and counterstained with hematoxylin. Quantification was performed using H‐score or percent‐positive metrics from multiple random fields per tumor by automated software. Inclusion criteria for fields were predefined.

### 4.22 Co‐Immunoprecipitation

For co‐immunoprecipitation, nuclear extracts were prepared in non‐denaturing buffer. Lysates were incubated with antibody against WDR5 overnight at 4°C and captured with protein A/G beads. Beads were washed under defined salt conditions, and bound proteins were eluted for immunoblotting.

### 4.23 In vivo Xenograft Studies, IVIS Imaging, and Tissue Processing

All animal procedures were approved by the relevant institutional animal care and use committee and were performed in accordance with institutional guidelines. Reporting followed the available ARRIVE‐style information from the study records. Female NOG mice were implanted subcutaneously with luciferase‐expressing gastric cancer cells suspended in PBS/Matrigel. Randomization was performed when tumors reached approximately 100 mm3 or when IVIS signal stabilized, as shown in Figure 6a. Tumors were measured by calipers 2–3 times per week; volume was calculated as (length x width^2)/2.

Mice were randomized to treatment groups when tumors reached approximately 100 mm3 or when IVIS signal stabilized. IVIS acquisition, caliper measurement, and image quantification used preset acquisition settings, predefined measurement rules, and identical regions of interest across time points. Vehicle, SAHA, WINi, or WINi plus SAHA were administered during the treatment window shown in Figure 6a. Body weight was recorded at each measurement time point as an initial tolerability readout. No comprehensive toxicology panel was included in this dataset. For IVIS imaging, mice received D‐luciferin (150 mg/kg, intraperitoneal), were anesthetized with isoflurane, and were imaged at a fixed post‐injection time (10–15 min) using an IVIS Spectrum. Radiance was quantified with identical regions of interest across time points and normalized to baseline (Day 0).

At endpoint, tumors were excised, weighed, and split for (i) snap‐freezing for protein/RNA extraction and (ii) fixation in 10% neutral buffered formalin for histology. Exclusion criteria included tumors with ulceration, mice with >15% body weight loss, technical IVIS failures, or failure to meet prespecified randomization criteria; exclusions and exclusion reasons were recorded per group when applicable.

For the treatment‐withdrawal study shown in Figure 6k, treatment was stopped after the treatment‐on period indicated in the figure (Day 0–7), and tumor volumes were followed during the treatment‐off period through Day 28 using the same measurement schedule.

For the in vivo WDR5‐dependency experiment shown in Figure 6l, xenografts representing NTC, sgWDR5, WDR5‐WT‐rescue and WIN‐pocket‐mutant‐rescue backgrounds were assigned to vehicle or SAHA treatment. Tumor radiance was normalized within each genetic background to its corresponding vehicle group (= 1), and tumors were excised and weighed at endpoint.

### 4.24 Pharmacokinetics

For drug‐exposure measurements, blood and tumor samples were collected 1 h after dosing unless otherwise indicated. Plasma was separated by centrifugation, and tumors were homogenized in a defined buffer. SAHA and WINi concentrations were quantified by LC–MS/MS using analyte‐specific external calibration curves and internal standards. Figure 6m summarizes the displayed plasma and tumor exposure values for the labeled SAHA, WINi, and combination treatment arms.

### 4.25 Excess‐Over‐Additivity Analysis

To compare observed combination outcomes with an additive expectation, expected additive values were computed from the single‐agent arms using the analysis model. The corresponding observed combination values were derived for tumor‐volume AUC and the radiance metric shown in Figure 6n. Confidence intervals were estimated by bootstrap resampling across animals.

### 4.26 Bioinformatics and Computational Analysis

Sequencing data were processed using a reproducible workflow. For RNA‐seq, STAR was used for alignment, featureCounts for quantification, and DESeq2 for differential expression with shrinkage of log2 fold changes where applicable. For ATAC‐seq and H3K4me3 profiling, alignments were performed with bowtie2 and signal tracks were represented as normalized bigWigs. Standard TSS‐centered matrices were generated relative to gene annotations, whereas the updated Figures 3g,h and 5a,b panels used the reciprocal held‐out, data‐derived‐center workflows described above. Genome‐track displays used the corresponding normalized bigWig files.

GSEA was performed using MSigDB gene sets with fgsea. Unless stated, genes with low counts were filtered by standard DESeq2 criteria. All analyses were conducted in R and Python with set random seeds for stochastic steps.

### 4.27 Statistical Analysis

No statistical methods were used to predetermine sample sizes. For the displayed in vivo panels, *n* = 3 mice per group unless otherwise indicated in Source Data. Tumor‐volume and IVIS time‐course data were analyzed using repeated‐measures ANOVA models with multiple‐comparison correction; endpoint tumor weight, body weight, IHC and biochemical quantifications were analyzed with two‐tailed tests or ANOVA as appropriate. Data were analyzed in Prism and/or R.

For two‐group comparisons, two‐tailed unpaired Student's t‐tests were used when normality and variance assumptions were met; otherwise, Mann‐Whitney tests were applied. For ≥3 groups, one‐way or two‐way ANOVA with appropriate multiple‐comparison corrections (Tukey or Sidak) was used. For time‐course data (tumor volume, IVIS radiance, regrowth curves), mixed‐effects models or repeated‐measures ANOVA were applied where appropriate, with subject (mouse or biological replicate) as a random effect. Endpoint tumor weight, IHC quantification, and tolerability readouts were analyzed using the test specified for each panel, with multiple‐comparison correction where applicable. (Supporting Information).

Correlation coefficients were computed using Pearson's r unless distributions were non‐normal, in which case Spearman's ρ was used. Multiple‐testing correction used Benjamini–Hochberg FDR. Significance thresholds were set at FDR <0.05 for omics and P <0.05 for targeted assays unless otherwise specified. Exact P values, test names, and n values are reported in figure legends or source data.

## Author Contributions

**Jintian Song**: conceptualization, investigation. **Hui Yu**: validation, visualization. **Jianping Jiang**: writing – Review Editing project Administration, formal Analysis, software, investigation, funding Acquisition. **Zhihui Jiang**: conceptualization, investigation, visualization, project Administration, formal Analysis. **Zhongshi Hong**: software, formal Analysis. **Xu Zhang**: investigation, project Administration. **Luchuan Chen**: methodology, funding Acquisition. **Shenghong Wei**: visualization, funding Acquisition. **Yi Wang**: conceptualization, validation; visualization.

## Funding

This research was sponsored by the Natural Science Foundation of Fujian Provincial Health Technology Project(Grant number:2024GGA047), the Startup Fund for Scientific Research, Fujian Medical University (Grant number: 2023QH1527), the Fujian Cancer Hospital High‐Level Talent Training Project (Grant number: 2023YNG08), the IIT Funds of Fujian Cancer Hospital (Grant number: 202413002), and the Fujian Cancer Hospital 2026 Outstanding Young Talent Program.

## Ethics Statement

The animal study protocol was reviewed and approved by the Experimental Animal Ethics Committee of the Laboratory Animal Ethics Committee of the Wenzhou Institute, University of Chinese Academy of Sciences (approval no. WIUCAS‐JJ‐25062603). All animal procedures were conducted in accordance with the institutional guidelines for animal welfare and the ethical principles for the care and use of laboratory animals.

## Consent

The authors have nothing to report.

## Conflicts of Interest

The authors declare no conflicts of interest.

## Supporting information




**Supporting File**: advs78255‐sup‐0001‐SuppMat.pdf

## Data Availability

The data that support the findings of this study are available from the corresponding author upon reasonable request.
