# Supplementary material for: Disabling COMPASS Promoter Licensing Shifts HDAC‐Inhibited Gastric Cancer Toward Sustained Transcriptional Suppression During Recovery
Source: Adv Sci (Weinh). 2026 Oct 15:e78255. Online ahead of print. doi: 10.1002/advs.78255 (PMC13672680; doi:10.1002/advs.78255)
Supplement: Supplementary file 1 [file ADVS-9999-e78255-s001.pdf]

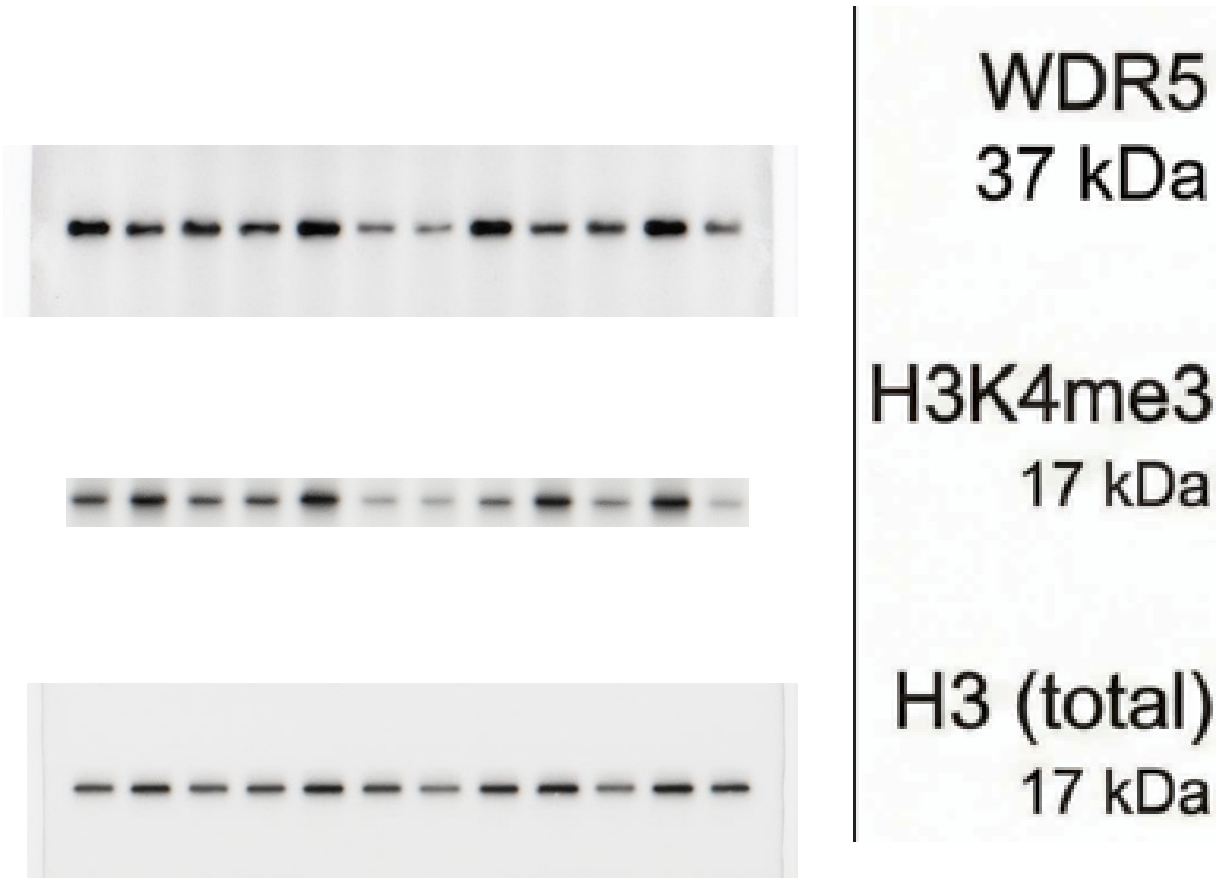
WDR5
37 kDa
H3K4me3
17 kDa
H3 (total)
17 kDa

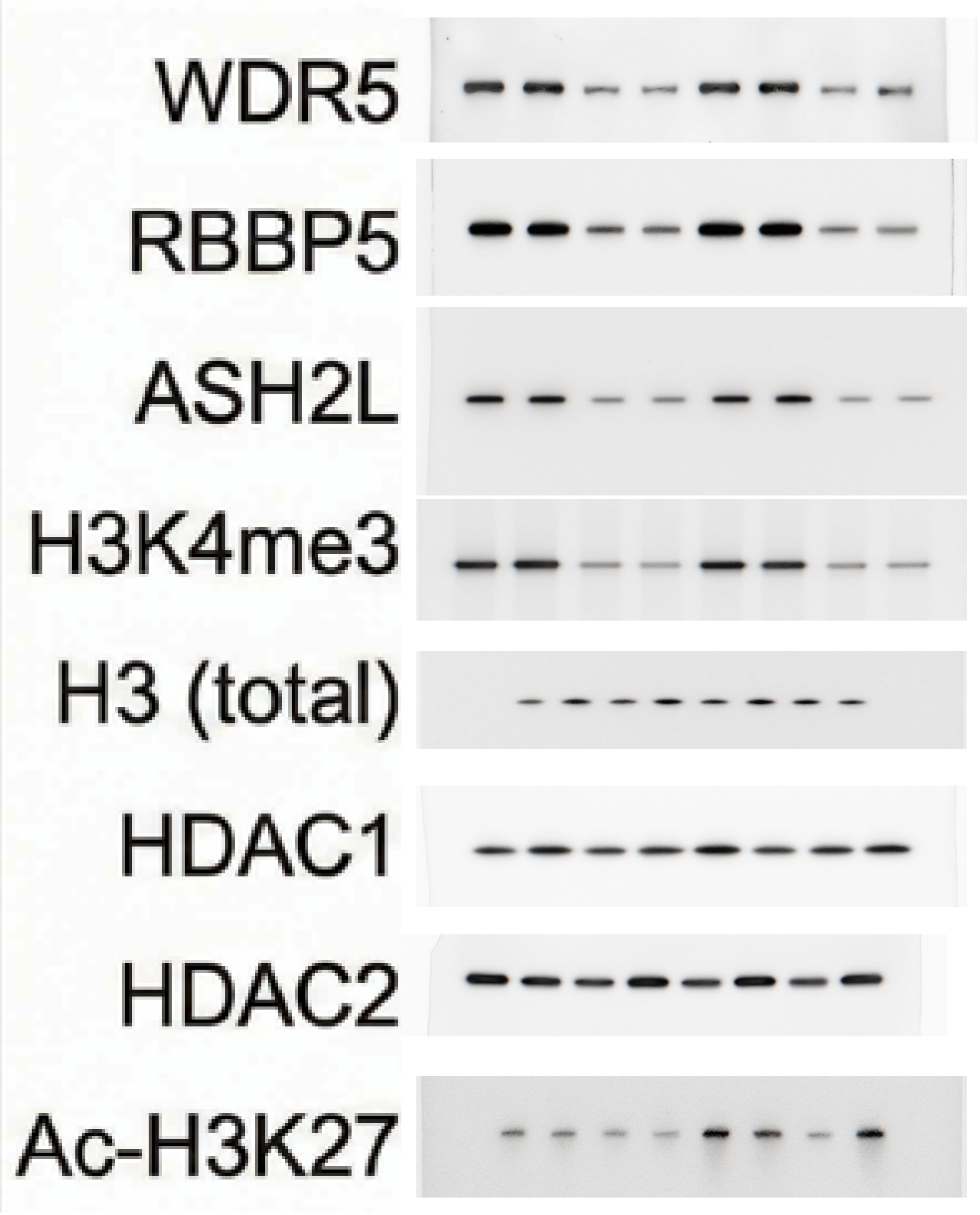
WDR5
RBBP5
ASH2L
H3K4me3
H3 (total)
HDAC1
HDAC2
Ac-H3K27

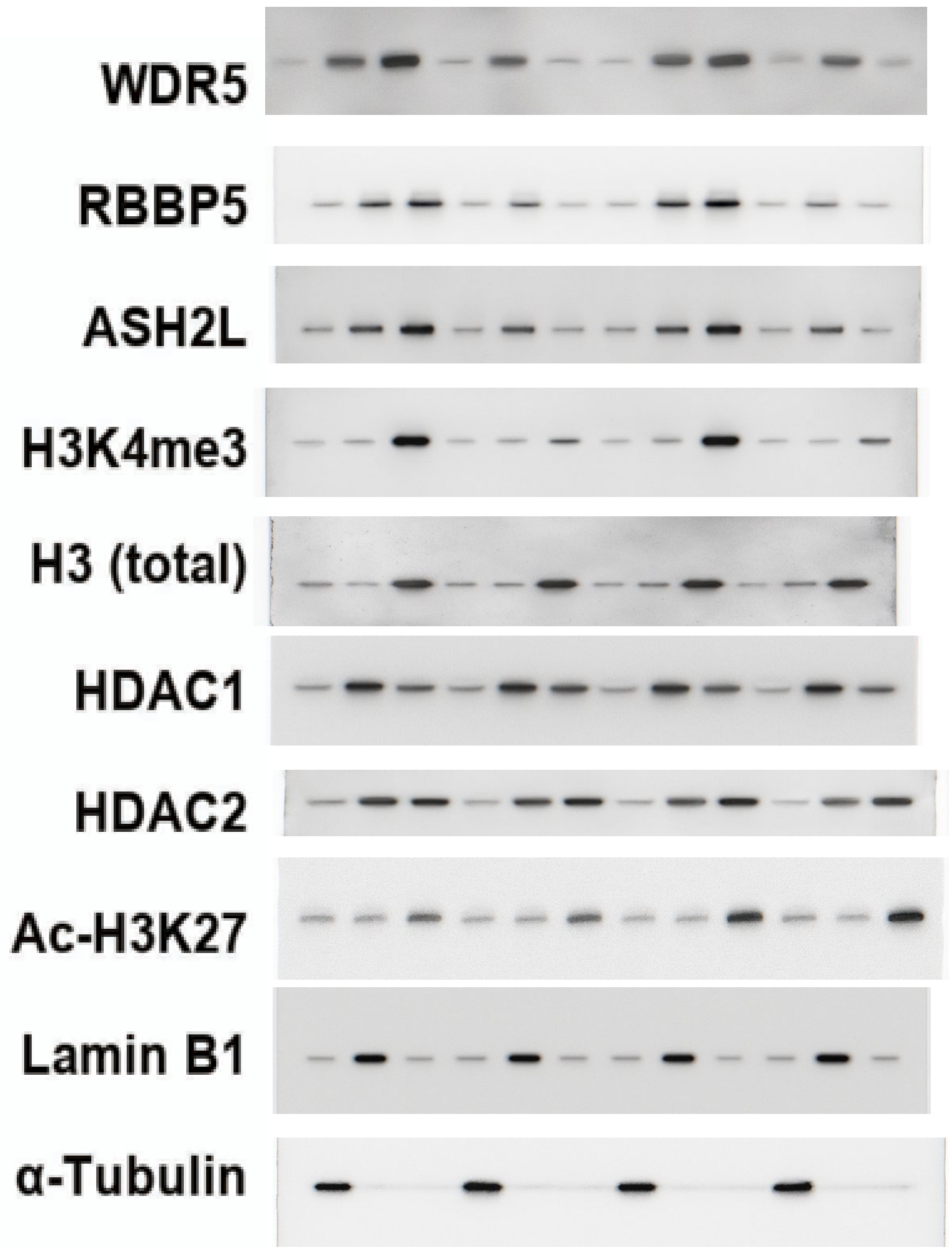
WDR5
RBBP5
ASH2L
H3K4me3
H3 (total)
HDAC1
HDAC2
Ac-H3K27
Lamin B1
α-Tubulin

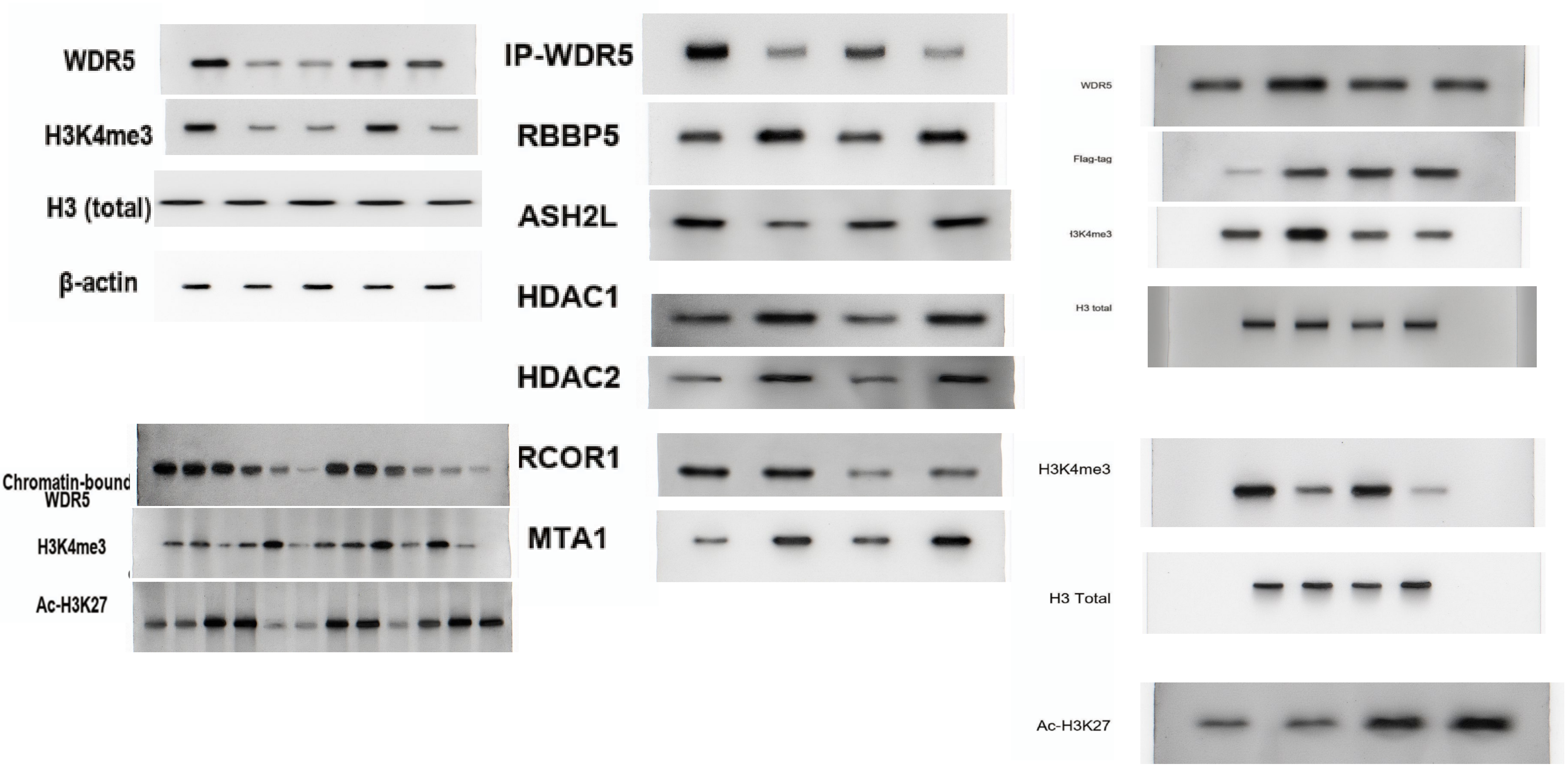
WDR5
H3K4me3
H3 (total)
β-actin
Chromatin-bound WDR5
H3K4me3
Ac-H3K27
IP-WDR5
RBBP5
ASH2L
HDAC1
HDAC2
RCOR1
MTA1
WDR5
Flag-tag
H3K4me3
H3 total
H3K4me3
H3 Total
Ac-H3K27

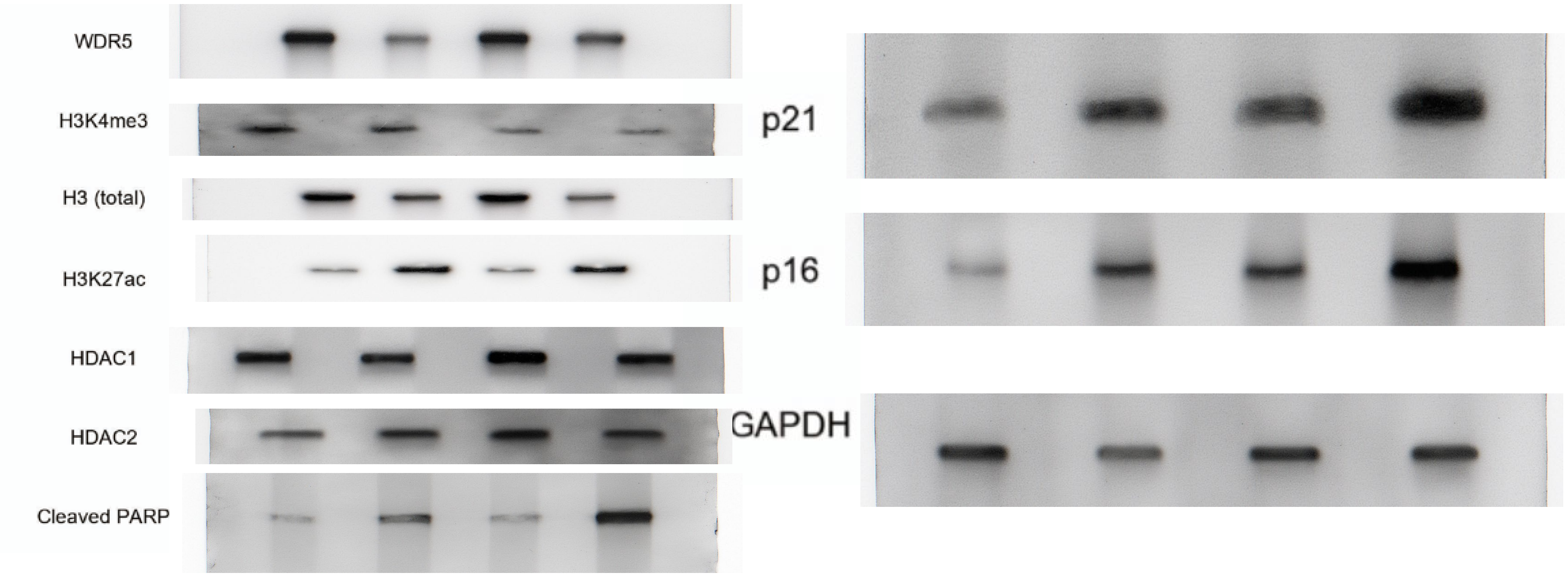
WDR5
H3K4me3
H3 (total)
H3K27ac
HDAC1
HDAC2
Cleaved PARP
p21
p16
GAPDH
